# Physical Exercise vs. Metformin to Improve Delivery- and Newborn-Related Outcomes Among Pregnant Women With Overweight: A Network Meta-Analysis

**DOI:** 10.3389/fmed.2021.796009

**Published:** 2021-12-09

**Authors:** Carlos Pascual-Morena, Iván Cavero-Redondo, Celia Álvarez-Bueno, José Alberto Martínez-Hortelano, Sara Reina-Gutiérrez, Alicia Saz-Lara, Sergio Núñez de Arenas-Arroyo, Vicente Martínez-Vizcaíno

**Affiliations:** ^1^Health and Social Research Center, Universidad de Castilla—La Mancha, Cuenca, Spain; ^2^Rehabilitation in Health Research Center (CIRES), Universidad de las Américas, Santiago, Chile; ^3^Universidad Politécnica y Artística del Paraguay, Asunción, Paraguay; ^4^Guadalajara University Hospital, Health Service of Castilla-La Mancha (SESCAM), Guadalajara, Spain; ^5^Facultad de Ciencias de la Salud, Universidad Autónoma de Chile, Talca, Chile

**Keywords:** pregnancy, exercise, metformin, overweight, obesity, systematic review, network meta-analysis

## Abstract

**Background:** Overweight/obesity is associated with the risk of delivery- and newborn-related complications in pregnancy. Interventions such as exercise or metformin could reduce the risk of these complications.

**Objective:** To estimate and compare the effects of different types of exercise interventions (i.e., aerobic, resistance, combined exercise) and metformin on delivery- and newborn-related outcomes among pregnant women with overweight/obesity.

**Methods:** MEDLINE, Scopus, Web of Science, Cochrane Library databases and the gray literature were searched from inception to September 2021. This systematic review was registered in PROSPERO (CDR: 42019121715). Randomized controlled trials (RCTs) of metformin or an exercise intervention aimed at preventing cesarean section, preterm birth, macrosomia, or birth weight among pregnant women with overweight/obesity were included. Random effects meta-analyses and frequentist network meta-analyses (NMA) were conducted for each outcome.

**Results:** Fifteen RCTs were included. In the NMA, metformin reduced the risk of cesarean section (RR = 0.66, 95% CI: 0.46, 0.95), combined exercise reduced the risk of macrosomia (RR = 0.37, 95% CI: 0.14, 0.95), and aerobic exercise reduced birth weight (mean difference = −96.66 g, 95% CI: −192.45, −0.88). In the subgroup among pregnant women with obesity, metformin reduced the risk of cesarean section (RR = 0.66, 95% CI: 0.45, 0.97).

**Conclusions:** Combined exercise could reduce the risk of macrosomia in pregnant women with overweight, whereas metformin could reduce the risk of cesarean section in pregnant women with obesity. However, previous evidence suggests a larger effect of physical exercise in other outcomes for this population group. Therefore, the medicalization of healthy pregnant women with obesity is not justified by the current evidence.

**Systematic Review Registration:** PROSPERO: CRD42019121715; https://www.crd.york.ac.uk/prospero/display_record.php?ID=CRD42019121715

## Introduction

Pre-pregnancy overweight and obesity are global public health problems that affect ~40% of women (1). The risk of numerous adverse deliveries and newborn events are increased by overweight and obesity, such as the risk of gestational diabetes mellitus, hypertensive disorders of pregnancy, macrosomia, preterm birth, or cesarean sections delivery (2–4). Furthermore, overweight and obesity also cause long-term health problems in offspring through epigenetic and microRNA interaction mechanisms, such as obesity or type II diabetes (5–7).

The rate of total cesarean sections is too high according to WHO recommendations, which estimate that only 10% of cesarean sections are actually necessary (8), but in the United States, 18.5% of deliveries in 2010 were by cesarean section (9). Preterm birth is defined as birth occurring before 37 weeks of gestation, and its incidence globally and in the United States is estimated to be approximately 10% (10). Additionally, macrosomia is usually defined as a birth weight greater than 4,000–4,500 g (depending on the guideline or author) (11), with an incidence of 9% in the United States (considering macrosomia as birth weight > 4,000 g) (12).

International guidelines recommend at least 30 mins of moderate-vigorous physical activity per day, including aerobic or combined (resistance and aerobic exercise) activity (13). It is not ruled out that exercise could have a protective effect on the development of fetal macrosomia and cesarean sections (14, 15). For preterm birth, in the past, exercise in pregnancy was discouraged because of theoretical risks. However, currently, some authors have even proposed that it is beneficial, but that is not without controversy (16). Metformin has also been proposed for pregnant women with gestational diabetes mellitus, polycystic ovary syndrome, and obesity. Despite being a safe drug and having some benefits in women with gestational diabetes mellitus or polycystic ovary syndrome, the benefits of prescribing metformin in non-diabetic women with obesity are unclear (17).

In a previous network meta-analysis (18), metformin reduced the risk of cesarean section. Moreover, a previous network meta-analysis by our group (19) showed that the type of exercise (i.e., aerobic, resistance, or combined) could determine the effect obtained, as observed with aerobic exercise and the risk of gestational diabetes mellitus. Therefore, the aim of this systematic review and network meta-analysis is to estimate the effect of metformin and different types of exercise on the development of delivery and newborn complications, including the risk of cesarean section, preterm birth, macrosomia, and birth weight among pregnant women with overweight/obesity.

## Methods

This systematic review and network meta-analysis was conducted according to the Cochrane Collaboration Handbook and the Preferred Reporting Items for Systematic Review incorporating Network Meta-analysis (PRISMA-NMA) (20, 21). The study protocol was registered in PROSPERO (registration number: CDR: 42019121715) and published elsewhere (22).

### Search Strategy

MEDLINE, Scopus, Web of Science, and Cochrane Library databases were searched from their inception to September 2021. We also reviewed clinicaltrials.gov, EudraCT, the gray literature, and the reference list of previous systematic reviews and articles included in this review. The databases searched, keywords, and additional information are detailed in Supplementary Appendix S1. The search and selection of studies was conducted independently by two reviewers (CP-M and CA-B), and disagreements were resolved by consensus or by a third reviewer (VM-V).

### Eligibility

The inclusion criteria were as follows: (1) type of study: randomized controlled trials; (2) type of participants: pregnant women with overweight or obesity; (3) type of interventions: structured exercise program (aerobic, resistance or combined exercises) or metformin treatment as the intervention, and (4) type of outcome assessment: delivery-related outcomes (i.e., risk ratio (RR) of cesarean section, RR of preterm birth) or newborn-related outcomes (i.e., RR of macrosomia, difference in mean birth weight). There was no language restriction.

The exclusion criteria were as follows: (1) type of studies: single-arm studies or non-randomized controlled trials; (2) type of participants: studies whose target population was exclusively women with pregestational insulin resistance, polycystic ovary syndrome, or other diseases that could affect the main outcomes; (3) type of intervention: dietary intervention as the primary cointervention, nutraceutical or dietary supplement interventions, or unstructured exercise intervention.

### Data Extraction

CP-M and IC-R extracted the data from the included studies according to the following predetermined information for each study: (1) reference, (2) country, (3) design), (4) participants (sample size, age, weight status), (5) intervention (type of intervention, frequency, length, intensity), (6) outcomes: risk of cesarean section, preterm birth, macrosomia, and/or birth weight.

### Categorization of Available Evidence

We used the original studies' classification to categorize body mass index as overweight or obese. When they did not report this categorization, the baseline body mass index was considered overweight (body mass index: 25–30 kg/m^2^) and obese (body mass index ≥ 30 kg/m^2^) (23).

Exercise was defined as a subset of structured and repetitive physical activity with the objective of improving or maintaining physical fitness (24). We classified exercise interventions into three categories: (1) aerobic exercise, (2) resistance training, and (3) combined exercises. Aerobic exercises are aimed at increasing energy expenditure and include walking, running, cycling, jogging, swimming, or interval exercise. Strength training was aimed at increasing muscle strength and included exercises with elastic bands or dumbbells, among others. Combined exercise includes, alternately or in combination, aerobic and strength exercises.

The intensity of the exercise intervention was reported by the authors and was classified as vigorous, moderate-vigorous, moderate, light-moderate, or light. When the authors did not report intensity, we used the criteria from the American College of Sports Medicine guidelines to estimate it (25–27) based on the percentage of maximum heart rate, percentage of heart rate, percentage of maximum oxygen uptake, or rating of perceived exertion reported by the studies.

### Risk of Bias Assessment

The risk of bias assessment of the included randomized controlled trials was conducted by two researchers (CP-M and IC-R) using the Cochrane Collaboration's tool for assessing risk of bias (28). This tool assesses the risk of bias of six domains: (1) randomization process, (2) assignment to intervention, (3) adherence to intervention, (4) missing outcome data, (5) measurement of the outcome, (6) selection of the reported result. Finally, the overall bias is scored as high/low/moderate (some concerns) risk of bias. Any disagreements were resolved by consensus or by a third reviewer (VM-V).

### Grading the Quality of Evidence

We used the Grading of Recommendations, Assessment, Development and Evaluation tool to assess the quality of evidence and make recommendations (29, 30). Each outcome obtained a high, moderate, low, or very low level of evidence, depending on several domains pre-established by the tool.

### Data Synthesis

We summarized the clinical trials in an *ad hoc* table describing the types of direct and indirect comparisons. Our network meta-analyses were conducted following the PRISMA-NMA statement (21).

We used a network geometry graph to assess the robustness of the evidence. The size of the nodes was proportional to the sample size of the trials, the thickness of the continuous line connecting the nodes was proportional to the sample size in trials directly comparing the two treatments, and the dashed lines represented indirect comparisons (31, 32).

Consistency was assessed by checking whether intervention effects estimated from indirect comparisons were consistent with those estimated from direct comparisons. We conducted the Wald test, and due to the low statistical power, the side-splitting assessment was also used (33). For statistically significant effects, the number needed to treat was estimated using the risk ratio obtained in the network meta-analysis and the basal risk.

We conducted a standard meta-analysis and frequentist network meta-analysis for direct and indirect comparisons between interventions and control groups (34, 35). Statistical heterogeneity was examined by the I^2^ statistic, was classified as not important (<40%), moderate (30–60%), substantial (50–90%), or considerable (>75%) (20). The *p*-values were also considered. The *τ*^2^ statistic was calculated to determine the size and clinical relevance of the heterogeneity. *τ*^2^ = 0.04 was considered a low, 0.14 was considered moderate, and 0.40 was considered a substantial degree of clinical relevance of the heterogeneity (36, 37). We displayed these results by creating both forest plots and a league table.

The transitivity requirement was assessed, checking that the synthesis of direct comparisons of two treatments had been conducted in similar studies on the most important clinical and methodological characteristics, including basal age and basal body mass index (38).

We conducted a relative ranking of treatments to identify superiority (31), and we estimated the surface under the cumulative ranking for each intervention (32).

We conducted a sensitivity analysis through a subgroup analysis with pregnant women with obesity using a random effects meta-analysis and a frequentist network meta-analysis for each outcome. Additionally, we conducted a sensitivity reanalysis from a Bayesian perspective.

To rule out a dependent effect of maternal body mass index or maternal weight gain, meta-regressions were performed using the risk ratios as the dependent variable. Finally, we also performed random effects meta-regression models, using as independent variables age, body mass index, intensity of intervention (or dose of metformin), weekly exercise frequency, duration of exercise session, gestational age at baseline, length of intervention, and total number of exercise sessions.

Finally, we used a funnel plot to visually examine the symmetry criterion to determine the presence of bias due to the small study effect (39). We conducted all analyses in Stata 15.0 (Stata, College Station, Texas, United States).

### Modifications to the Initial Protocol

In the protocol, the target population included all pregnant women, and the inclusion of all types of trials. It was decided to limit it to pregnant women with overweight/obesity in randomized clinical trials to improve the transitivity principle and the quality of the final analyses. Finally, the protocol established the performance of Bayesian network meta-analysis. Subsequently, it was decided to conduct frequentist network meta-analyses and a sensitivity reanalysis using a Bayesian perspective.

## Results

**Figure 1:**
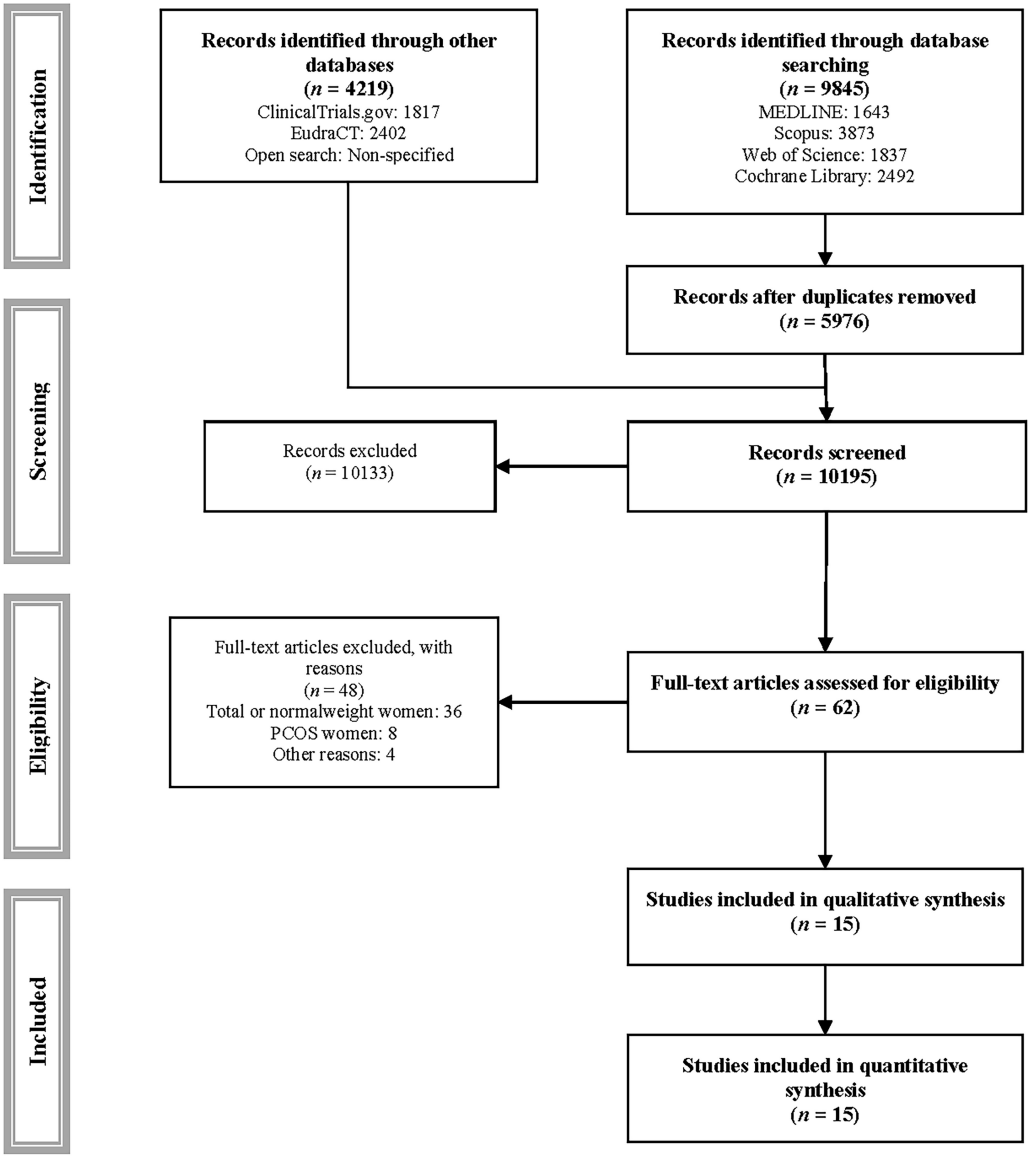
PRISMA flowchart of study selection.

Fifteen randomized controlled trials (40–54) were included in the analyses (Table 1, Figure 1, Supplementary Table S1), and 48 studies were excluded for various reasons (Supplementary Table S2). Of the included trials, 6 included pregnant women with overweight, and 13 pregnant women with obesity. The trials were conducted in 10 countries: 8 in Europe, including two in the Netherlands (48, 51), two in Spain (43, 49), two in the United Kingdom (52, 54) and one each in Ireland (45) and Norway (46), 5 in America, including three in Brazil (47, 50, 53), one in Canada (44) and one in the United States (40), one in Asia (China) (42), and one in Oceania (New Zealand) (41). A total of 2,759 pregnant women were included in the trials (412 in aerobic exercise, 1,021 in combined exercise, and 1,326 metformin interventions). Exercise frequency was two to five times per week, lasting 12–30 weeks. The dose of metformin was between 1,000 and 3,000 mg per day, lasting ~25 weeks. The details of the interventions are described in Supplementary Table S3.

**Table 1:** Characteristics of included trials.

**References**	**Country**	**Participants**						**Intervention**	**Outcomes**			
		**N_**T**_**	**N_**I**_**	**N_**C**_**	**Age_**I**_**	**Age_**C**_**	**Weight status**		**RR CS**	**RR PR**	**RR MA**	**BW**
Kong et al. (40)	United States	19	9	9	26.2 ± 2.6	27.3 ± 3.6	Overweight	Aerobic exercise	✓	✓	✓	✓
Kong et al. (40)	United States	18	9	10	28.6 ± 5.3	25.7 ± 4.0	Obesity	Aerobic exercise	✓	✓	✓	✓
Seneviratne et al. (41)	New Zealand	75	38	37	NA	NA	Obesity	Aerobic exercise	✓	✓	✓	✓
Wang et al. (42)	China	300	150	150	32.1 ± 4.6	32.5 ± 4.9	Overweight	Aerobic exercise	✓	✓	✓	✓
Barakat et al. (43)	Spain	168	90	78	–	–	Overweight	Combined exercise	–	✓	✓	–
Barakat et al. (43)	Spain	54	25	29	–	–	Obesity	Combined exercise	–	✓	✓	–
Bisson et al. (44)	Canada	50	25	25	30.5 ± 3.7	31.0 ± 4.0	Obesity	Combined exercise	✓	–	–	✓
Daly et al. (45)	Ireland	88	44	44	30.0 ± 5.1	29.4 ± 4.8	Obesity	Combined exercise	✓	✓	✓	✓
Garnæs et al. (46)	Norway	91	46	45	31.3 ± 3.8	31.4 ± 4.7	Obesity	Combined exercise	✓	✓	✓	✓
Nascimento et al. (47)	Brazil	82	40	42	29.7 ± 6.8	30.9 ± 5.9	Overweight/Obesity	Combined exercise	✓	–	–	✓
Oostdam et al. (48)	Netherlands	121	62	59	30.8 ± 5.2	30.1 ± 4.5	Obesity	Combined exercise	✓	–	–	✓
Ruiz et al. (49)	Spain	275	146	129	–	–	Overweight/Obesity	Combined exercise	✓	✓	✓	✓
Santos et al. (50)	Brazil	92	46	46	26.0 ± 3.4	28.6 ± 5.9	Overweight	Combined exercise	–	✓	–	✓
Brink et al. (51)	Netherlands	49	24	25	29.3 ± 5.2	30.7 ± 5.2	Obesity	Metformin	✓	✓	–	✓
Chiswick et al. (52)	United Kingdom	449	226	223	28.7 ± 5.8	28.9 ± 5.1	Obesity	Metformin	✓	✓	–	✓
Nascimento et al. (53)	Brazil	378	189	189	28.6 ± 6.2	29.6 ± 6.1	Obesity	Metformin	✓	✓	–	–
Syngelaki et al. (54)	United Kingdom	450	225	225	32.9	30.8	Obesity	Metformin	✓	✓	✓	–

*N_T_, sample size; N_I_, sample size in intervention group; N_C_, sample size in control group; Age_I_, mean age in intervention group; Age_C_, mean age in control group; RR CS, risk ratio of cesarean section; RR PR, risk ratio of preterm birth; RR MA, risk ratio of macrosomia; BW, birthweight (mean differences)*.

### Cesarean Section

**Figure 2:**
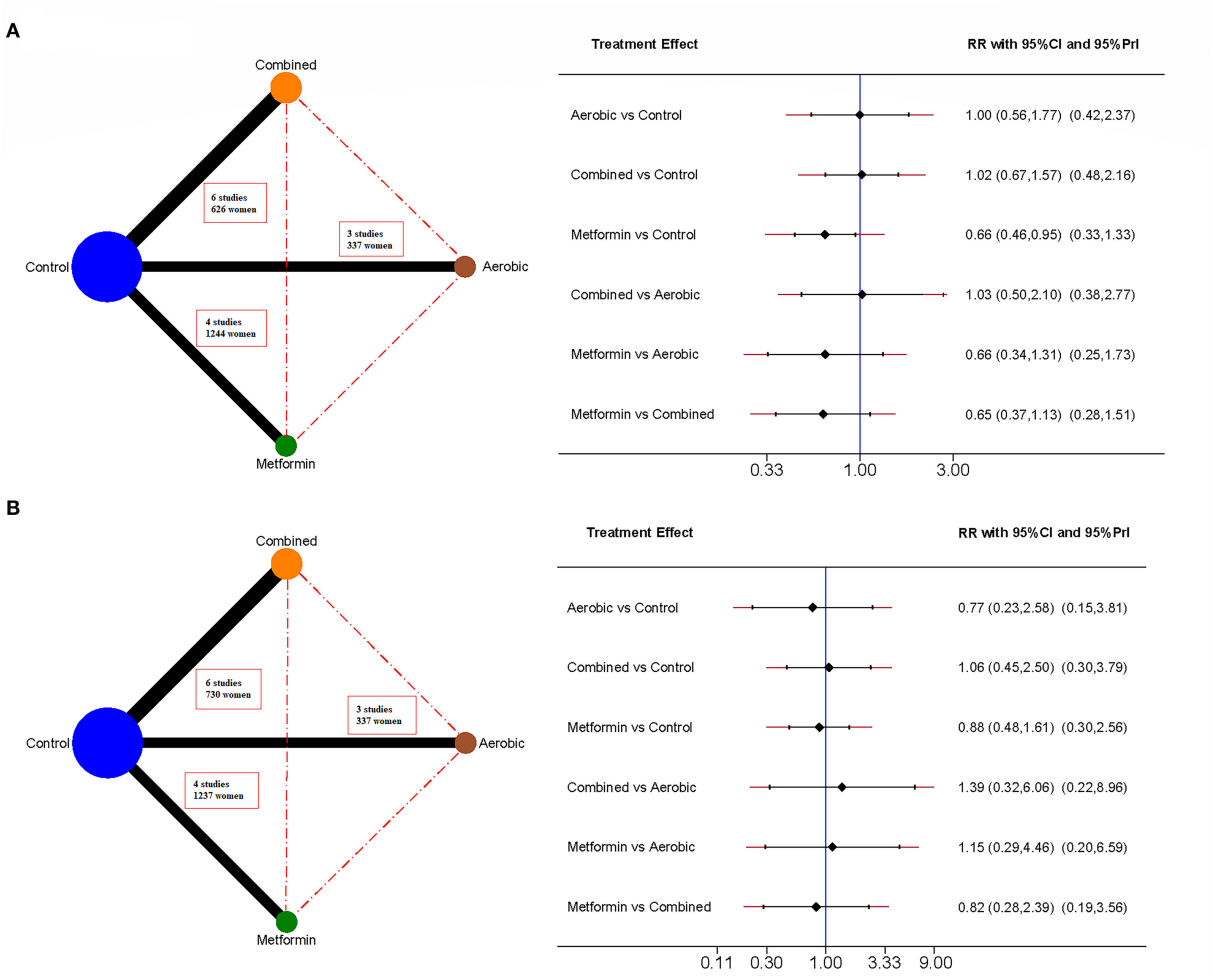
Network meta-analyses for delivery-related outcomes. It includes cesarean section **(A)** and preterm birth **(B)**. The network mapping is shown on the left, and the network meta-analysis estimates on the right, measured as risk ratio (RR) and 95% confidence interval (95% CI).

Figure 2A, Supplementary Table S4A, and Supplementary Figure S1A show the standard pairwise comparisons (upper diagonal) and the network meta-analysis (under diagonal). Metformin reduced the risk of cesarean section in pairwise comparisons and in the network meta-analysis (RR = 0.79, 95% CI: 0.63, 0.99, and RR = 0.66, 95% CI: 0.46, 0.95, respectively). The number needed to treat of metformin was 8 women to prevent one case.

### Preterm Birth

Figure 2B, Supplementary Table S4B, and Supplementary Figure S1B show that no intervention had a statistically significant effect on either the pairwise comparisons or the network meta-analysis.

### Macrosomia

**Figure 3:**
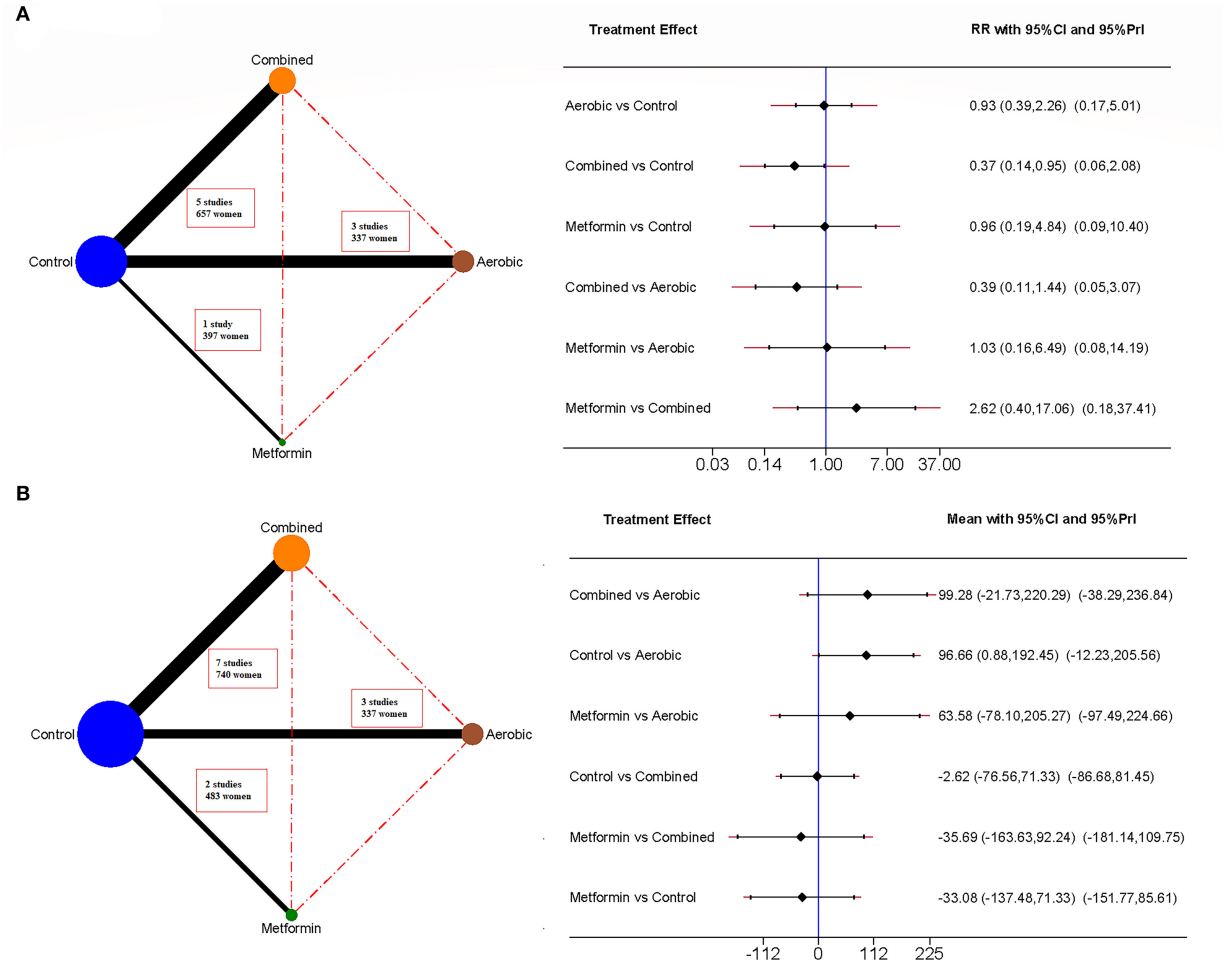
Network meta-analyses for newborn-related outcomes. It includes macrosomia **(A)** and birth weight **(B)**. The network mapping is shown on the left, and the network meta-analysis estimates on the right, measured as risk ratio (RR) and 95% confidence interval (95% CI).

Figure 3A, Supplementary Table S4C, and Supplementary Figure S1C show that combined exercise reduced the risk of macrosomia in the network meta-analysis estimates (RR = 0.37, 95% CI: 0.14, 0.95). The number needed to treat of the combined exercise was 12 women to prevent one case.

### Birth Weight

Figure 3B, Supplementary Table S4D, and Supplementary Figure S1D show that aerobic exercise reduces birth weight in the network meta-analysis (Mean Difference = −96.66 g, 95% CI: −192.45, −0.88).

### Risk of Bias

According to the Cochrane Collaboration's tool for assessing risk of bias, 10 out 15 (66.7%) showed a high risk of bias for overall bias, and five (33.3%) showed some concerns. By domain, 13.3% of the studies showed high risk for assignment to intervention, 66.7% showed some concerns, 46.7% showed high risk, 20.0% showed some concerns for adhering to intervention, 20.0% showed high risk, 26.7% showed some concerns for missing outcome data, and 66.7% showed some concerns for measurement of the outcome. No significant risk of bias was detected for the randomization process or for selection of the reported results. The total risk of bias is shown in the Supplementary Figure S2.

### Grades of Recommendation, Assessment, Development, and Evaluation

According to the Grading of Recommendations, Assessment, Development and Evaluation tool, all interventions showed very low certainty for all outcomes. Only the metformin intervention showed low certainty for the risk of cesarean section. The most affected domains were risk of bias, inconsistency, indirectness and imprecision. The complete assessment is detailed in Supplementary Table S5.

### Transitivity

There were no statistically significant differences in baseline age or body mass index between the two interventions for cesarean section and birth weight. There were statistically significant differences between the baseline body mass index of the intervention groups for preterm birth, with the baseline body mass index of the metformin intervention being higher than the exercise interventions. There were insufficient data to conduct a transitivity analysis for the risk of macrosomia. The complete assessment is detailed in Supplementary Table S6.

### Probabilities

**Figure 4:**
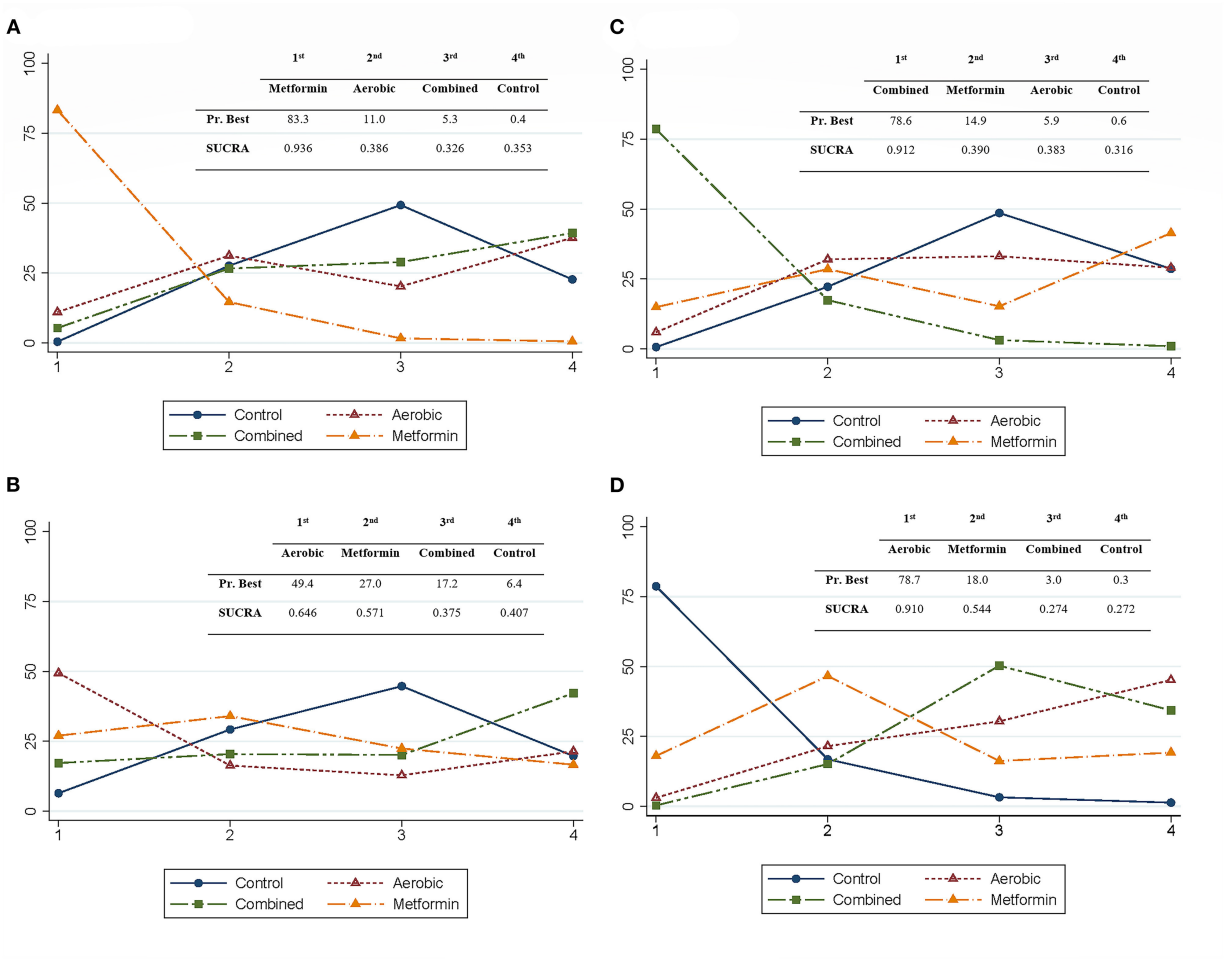
Relative rankings of treatments for delivery- and newborn-related outcomes. It includes cesarean section **(A)**, preterm birth **(B)**, macrosomia **(C)**, and birth weight **(D)**. Each image shows the rankogram, and a summarized table of the probability of being the best intervention (PrBest) and the surface under the cumulative ranking (SUCRA).

The Metformin intervention showed the highest probability of being the best intervention for preventing cesarean section (Probability of best intervention = 83.3%, surface under the cumulative ranking = 0.936), combined exercise was best for preventing macrosomia (Probability of best intervention = 78.6%, surface under the cumulative ranking = 0.912), and aerobic exercise was best for reducing birth weight (Probability of best intervention = 78.7%, surface under the cumulative ranking = 0.910). No intervention showed a high probability of preventing preterm birth (Figure 4, Supplementary Figure S3).

### Subgroup Analysis

Subgroup analysis examining only pregnant women with obesity showed no effect of any intervention to prevent preterm birth or macrosomia or to modify birth weight, but metformin significantly reduced cesarean sections (RR = 0.79, 95% CI: 0.63, 0.99, and RR = 0.66, 95% CI: 0.45, 0.97, in the pairwise comparisons and the network meta-analysis, respectively). The number needed to treat of metformin was 8 women to prevent one case. The complete subgroup analysis is detailed in Supplementary Table S7, Supplementary Figure S4.

### Sensitivity Analysis With Bayesian Methods

The sensitivity reanalysis using Bayesian methods did not produce statistically different results from the frequentist analyses.

### Meta-Regressions Models

There was no statistically significant association between body mass index or maternal weight gain and the included outcomes (Supplementary Table S8). No statistically significant association was observed for the variables studied for each intervention (Supplementary Table S9).

### Heterogeneity and Publication Bias

Combined exercise showed moderate heterogeneity for the risk of macrosomia (I^2^ = 53.48%) and metformin showed moderate heterogeneity for the risk of cesarean sections and birth weight (I^2^ = 55.24% and I^2^ = 46.71%, respectively), but the rest of the interventions did not show statistically significant heterogeneity in the studied outcomes. Aerobic and combined exercise showed moderate and substantial degrees of clinical relevance in the heterogeneity for the risk of macrosomia (τ^2^ = 0.11 and τ^2^ = 0.88, respectively), but the remaining interventions had a low degree of clinical relevance of the heterogeneity (Supplementary Figure S1). There was no evidence of publication bias in funnel plot asymmetry for any outcome (Supplementary Figure S5).

## Discussion

### Main Findings

Metformin in women with overweight/obesity was found to reduce the risk of cesarean section by 34% with a number needed to treat of 8, and combined exercise reduced the risk of macrosomia by 63%, with a number needed to treat of 12. Additionally, aerobic exercise reduced birthweight by 96.7 g. No effect was found on the risk of preterm birth for any intervention. No statistically significant association was observed in the meta-regressions. Finally, the study in subgroups of women with obesity confirmed the effect of metformin in reducing the risk of cesarean section.

### Interpretation

Regarding the cesarean section, our results confirm the findings of the previous network meta-analysis (18), in contrast to previous meta-analyses (55–57). Thus, in our study, metformin reduced the incidence by 34%. The observed effect is due to the inclusion of new studies with respect to previous meta-analyses, in which there was a non-significant trend to benefit. Interestingly, the meta-regressions did not show the effect of covariates such as dose and length of the intervention. Moreover, considering the dose and length of the individual studies with their risk ratios, there does not appear to be a dose-response association, which suggests that at moderate doses the desired effect could be achieved. The mechanism by which metformin reduces the risk of cesarean section is also unclear. Considering that body mass index is associated with birth weight and risk of cesarean section, the hypothesis that obesity and excessive maternal weight gain increases the deposition of fatty tissue in the maternal pelvis and increases birth weight, causing obstructed labor, is attractive (58). This hypothesis is supported by the reduction of maternal weight gain with metformin (19). However, in the meta-regressions, no association was observed between the maternal weight gain and the risk of cesarean section. In addition to the above, combined exercise, which reduces the incidence of macrosomia, had no effect on the risk of cesarean section. Another hypothesis is the possibility that metformin improves lipid profiles, including cholesterol, low-density lipoprotein and very low-density lipoprotein, which may negatively affect the contractility of the myometrium because of alterations in the fluidity and viscosity of cell membranes, which in turn alter the function of the calcium in muscle contraction (58), as well as the increase in glycogen stores in myometrial cells necessary to perform vigorous contractions (59). However, these two hypotheses have not been confirmed (58, 59), requiring future research.

Our results showed a reduction in the risk of macrosomia and birth weight with combined and aerobic exercise, respectively, which contrasts with the results of two previous meta-analyses (60, 61) which found no effect. These results pose a challenge, since the same type of intervention that decreased birth weight did not reduce macrosomia, and vice versa. There are several mechanisms that could explain it. The Pederson theory (62) established that maternal hyperglycaemia caused fetal hyperinsulinemia, with increased fetal weight and macrosomia. Although some effect cannot be ruled out, the lack of effect of metformin in these outcomes rules out that it is the main mechanism. In fact, exercise also improves the glycaemic profile (63–66), which does not necessarily translate into an improvement in fetal weight. Some authors (67) have recently proposed that the decrease in body fat with exercise is not directly because of the oxidation of fatty acids during exercise sessions, but to the uptake of fatty acids after exercise to repair tissue damage, which is especially the case with anaerobic or high intensity sessions. Thus, combined exercise, with the inclusion of strength exercises, could deprive the fetus of excess energy from fatty acids and reduce the risk of macrosomia in fetuses predisposed to it. The reason that aerobic exercise reduced birth weight was due to one trial (42) using moderate-vigorous intensity. At this intensity, in addition to having a possible anaerobic component and tissue damage, it could increase catecholamine levels causing lipolysis (67), and decrease uterine blood flow depending on time and intensity (66).

As expected, in the study by subgroups among pregnant women with obesity, the effect of metformin was maintained because of the inclusion of the same studies. However, no effect of combined exercise was found on the risk of macrosomia. In addition to the scarcity of studies, the exclusion of two inputs (43, 49) for not exclusively including women with obesity reduced the effect obtained, probably due to the high adherence of the participants in these two trials compared to other included trials. Finally, no effect on birthweight was observed in obese women, which was also expected, because of the exclusion of the trial with the greatest effect (42), suggesting the importance of intensity for this outcome.

Although there was variability in the cohorts of women and the interventions (i.e., body mass index, metformin dose, exercise intensity, length of interventions), we attempted to control by assessing the transitivity principle, meta-regressions, and subgroup analyses to provide consistent evidence to aid decision-making. First, the most interesting result is the effect of the combined exercise on the risk of macrosomia. Based on one study (42), international guidelines (13) suggest that aerobic exercise could reduce the incidence of macrosomia in pregnant women with overweight. Although the scarcity of studies does not allow us to reject this hypothesis, our study shows that the most effective exercise is combined, at light-moderate to moderate intensity, 50–60 mins per session, 3 times per week. Second, it is also suggested (13) that exercise could reduce the risk of preterm birth in women with overweight or obesity. Our study could not replicate these findings, however, it was shown to be safe, this being a relevant aspect due to the traditional fear of recommending exercise during pregnancy. Third, the effect of exercise on the risk of cesarean section pointed out by other authors (13, 68) could not be confirmed either. This is probably due to the inclusion of exclusively women with overweight and the lack of effect it may have on this outcome in this population. Fourth, metformin had no effect on the risk of macrosomia, which was interesting, but it did have a significant effect on the risk of cesarean section. Since metformin could have some effect on maternal weight gain and cesarean section, and it appears to be safe for newborns (19, 56), its use in specific cases and with a thorough assessment of the benefit-risk profile cannot be ruled out. However, the lack of effect on other outcomes, the low quality of the evidence (GRADE) and the caution in the administration of drugs during pregnancy do not allow to recommend a generalized medicalization of healthy pregnant women with obesity.

### Limitations

Some limitations should be acknowledged. First, the main limitation is related to the scarcity of studies, which could affect the effect estimates, especially for the aerobic exercise and metformin intervention, the statistical power of the network meta-analysis, the publication bias analysis, and the assessment of the transitivity requirement. Second, the lack of studies limited additional analysis (i.e., meta-regressions) by covariates or mediators that could determine the possible effect of length, frequency, or intensity of interventions. Third, only four outcomes were considered. This was because most trials only report these outcomes. Therefore, future research is needed to determine, by meta-analysis or NMA, the influence of exercise type and other covariates, on outcomes such as placental weight, gestational age at delivery, other birth weight categories (i.e., low and adequate birthweight) and Apgar score in 1 or 5 min. Fourth, neonatal glycemia is associated with long-term adverse events. However, the studies did not report these data, therefore, it was not possible to estimate the effect of the interventions on this parameter, nor the association between neonatal glycemia and the included outcomes. Fifth, for the diagnosis of macrosomia, the cut-off weight for categorization as macrosomia varied by author between 4,000 and 4,500 g, which could slightly affect the estimate of effect. Sixth, no differentiation was made between emergency and elective cesarean sections, which could affect the estimate of effect, although it is unlikely to have a statistically significant effect since both types of cesarean sections are associated with overweight/obesity. Seventh, no resistance exercise interventions were found, which can be a problem for understanding what effect adding resistance exercise to aerobic exercises can have. Eighth, we found a moderate to high risk of bias in most studies, with the domains 'adhering to intervention' and 'missing outcome data' being the ones that could most affect the effect estimates in our analyses. Nineth, although there were no statistically significant differences between the baseline age and body mass index for exercise and metformin on cesarean section and birth weight, overall there was high heterogeneity across studies. Tenth, we were unable to perform transitivity analysis for macrosomia due to lack of studies on metformin.

## Conclusions

Metformin reduces the risk of cesarean section in pregnant women with obesity, and combined exercise reduces the risk of fetal macrosomia in pregnant women with overweight/obesity. Aerobic exercise could also reduce birth weight. Additionally, exercise was safe for the risk of preterm birth, something that has been debated for decades. The meta-regressions were limited by the number of included studies, and therefore, further research is needed to determine the effect of the length, frequency, and intensity of each type of exercise, and the length and dosage of metformin, on the risk of the outcomes studied. Considering the limitations of the study and the quality of the evidence, the systematic medicalization of pregnancy among women with overweight/obesity is not justified. However, it is highly recommended that women without exercise contraindications perform structured exercise, including aerobic and strength exercises, and achieve high adherence.

## Data Availability Statement

The original contributions presented in the study are included in the article/Supplementary Material, further inquiries can be directed to the corresponding author.

## Author Contributions

CP-M and VM-V conceptualized the study. CP-M, IC-R, and CA-B planned and carried out the study, data curation, and investigation. CP-M, SR-G, JAM-H, AS-L, and SN-A-A carried out the formal analysis. CP-M, IC-R, and VM-V wrote the manuscript. VM-V provided the funding. All authors reviewed the manuscript.

## Funding

This study was funded by the Consejería de Educación, Cultura y Deportes—Junta de Comunidades de Castilla-La Mancha and European Regional Development Fund (SBPLY/17/180501/000533). CP-M was supported by a grant from the Universidad de Castilla-La Mancha(2018-CPUCLM-7939).

## Conflict of Interest

The authors declare that the research was conducted in the absence of any commercial or financial relationships that could be construed as a potential conflict of interest.

## Publisher's Note

All claims expressed in this article are solely those of the authors and do not necessarily represent those of their affiliated organizations, or those of the publisher, the editors and the reviewers. Any product that may be evaluated in this article, or claim that may be made by its manufacturer, is not guaranteed or endorsed by the publisher.
