# Supplementary material for: Physical Exercise vs. Metformin to Improve Delivery- and Newborn-Related Outcomes Among Pregnant Women With Overweight: A Network Meta-Analysis
Source: Front Med (Lausanne). 2021 Dec 9;8:796009. doi: 10.3389/fmed.2021.796009 (PMC8696129; doi:10.3389/fmed.2021.796009)
Supplement: Supplementary file 1 [file Data_Sheet_1.DOCX]

Supplementary Material

**Index**

**Table S1.** Direct estimates.

**Table S2.** Excluded trials with reasons.

**Table S3.** Characteristics of the interventions.

**Table S4.** Results for direct pairwise comparisons and network meta-analysis.

**Table S5.** Grades of Recommendation, Assessment, Development, and Evaluation.

**Table S6.** Transitivity analysis.

**Table S7.** Results for direct pairwise comparisons and network meta-analysis among pregnant women with obesity.

**Table S8.** Meta-regressions models by body mass index and maternal weight gain.

**Table S9.** Meta-regressions models by intervention.

**Figure S1.** Direct pairwise comparisons by outcome.

**Figure S2.** Risk of bias.

**Figure S3.** Cumulative probabilities by outcome.

**Figure S4.** Direct pairwise comparisons and network meta-analysis by outcome among pregnant women with obesity.

**Figure S5.** Funnel plot by outcome.

**Appendix S1.** Search Strategy.

**Table S1.** Direct estimates.

| Reference | Intervention | Caesarean section | | Preterm delivery | | Macrosomia | | Birthweight | |
| --- | --- | --- | --- | --- | --- | --- | --- | --- | --- |
|  |  | **RR** | **95% CI** | **RR** | **95% CI** | **RR** | **95% CI** | **MD** | **95% CI** |
| *Kong K et al (2014)-1* | Aerobic | 0.11 | (0.01, 1.80) | 0.33 | (0.02, 7.24) | 3.00 | (0.38, 23.68) | 170.00 | (-245.87, 585.87) |
| *Kong K et al (2014)-2* | Aerobic | 1.11 | (0.47, 2.60) | 1.10 | (0.02, 50.43) | 0.44 | (0.11, 1.75) | -400.00 | (-845.18, 45.18) |
| *Seneviratne SN et al (2016)* | Aerobic | 1.38 | (0.80, 2.40) | 2.00 | (0.19, 21.11) | 1.43 | (0.61, 3.35) | -16.00 | (-269.07, 237.07) |
| *Wang C et al (2017)* | Aerobic | 0.91 | (0.61, 1.34) | 0.61 | (0.15, 2.49) | 0.65 | (0.26, 1.61) | -112.19 | (-222.35, -2.03) |
| *Barakat R et al (2016)-1* | Combined | - | - | 0.47 | (0.20, 1.11) | 0.05 | (0.00, 0.87) | - | - |
| *Barakat R et al (2016)-2* | Combined | - | - | 1.74 | (0.32, 9.60) | 3.46 | (0.15, 81.36) | - | - |
| *Bisson M et al (2015)* | Combined | 1.00 | (0.45, 2.23) | - | - | - | - | 120.00 | (-104.92, 344.92) |
| *Daly N et al (2017)* | Combined | 0.86 | (0.50, 1.50) | 1.95 | (0.18, 20.77) | 2.93 | (0.12, 70.08) | -1.80 | (-218.51, 214.91) |
| *Garnæs KK et al (2017)* | Combined | 1.42 | (0.56, 3.59) | 4.74 | (0.24, 95.55) | 0.67 | (0.39, 1.14) | -193.00 | (-456.18, 70.18) |
| *Nascimento SL et al (2011)* | Combined | 0.91 | (0.67, 1.22) | - | - | - | - | 39.00 | (-244.54, 322.54) |
| *Oostdam N et al (2012)* | Combined | 0.99 | (0.41, 2.41) | - | - | - | - | 172.00 | (-54.09, 398.09) |
| *Ruiz JR et al (2013)* | Combined | 1.16 | (0.76, 1.76) | 1.77 | (0.33, 9.49) | 0.15 | (0.03, 0.65) | -36.00 | (-150.09, 78.09) |
| *Santos IA et al (2005)* | Combined | - | - | 1.89 | (0.18, 19.95) | **-** | **-** | -5.00 | (-241.09, 231.09) |
| *Brink HS et al (2018)* | Metformin | 0.73 | (0.33, 1.60) | 0.21 | (0.01, 4.12) | - | - | -199.80 | (-460.27, 60.67) |
| *Chiswick C et al (2015)* | Metformin | 0.87 | (0.66, 1.14) | 1.32 | (0.67, 2.59) | - | - | -1.00 | (-115.29, 113.29) |
| *Nascimento IB et al (2020)* | Metformin | 0.63 | (0.51, 0.78) | 1.09 | (0.44, 2.68) | - | - | - | - |
| *Syngelaki A et al (2016)* | Metformin | 0.94 | (0.74, 1.19) | 0.60 | (0.31, 1.16) | 0.97 | (0.28, 3.28) | - | - |

RR: Risk ratio; 95% CI: 95% confidence interval, MD: mean difference.

**Table S2.** Excluded trials with reasons.

| Reference | Intervention | Mean reason for exclusion |
| --- | --- | --- |
| *Calvalcante SR et al (2009) (1)* | Aerobic exercise | Total or normalweight women |
| *Carmen Carrascosa M et al (2021) (2)* | Aerobic exercise | Total or normalweight women |
| *Clapp JF et al (2000) (3)* | Aerobic exercise | Total or normalweight women |
| *de Oliveria Melo AS et al (2012) (4)* | Aerobic exercise | Total or normalweight women |
| *Ghodsi Z et al (2014) (5)* | Aerobic exercise | Total or normalweight women |
| *Guelfi KJ et al (2016) (6)* | Aerobic exercise | Total or normalweight women |
| *Hopkins SA et al (2011) (7)* | Aerobic exercise | Total or normalweight women |
| *Kasawara KT et al (2013) (8)* | Aerobic exercise | Other risk factors |
| *Khoram S et al (2019) (9)* | Aerobic exercise | Other risk factors |
| *Kihlstrand M et al (1999) (10)* | Aerobic exercise | Total or normalweight women |
| *Ko CW et al (2014) (11)* | Aerobic exercise | Total or normalweight women |
| *Labonte-Lemoyne E et al (2017) (12)* | Aerobic exercise | Total or normalweight women |
| *McDonald SM et al (2020) (13)* | Aerobic exercise | Total or normalweight women |
| *Marquez-Sterling S et al (2000) (14)* | Aerobic exercise | Total or normalweight women |
| *Sedaghati P et al (2007) (15)* | Aerobic exercise | Total or normalweight women |
| *Taniguchi C et al (2016) (16)* | Aerobic exercise | Total or normalweight women |
| *Tomic V et al (2013) (17)* | Aerobic exercise | Total or normalweight women |
| *Rakhshani A et al (2012) (18)* | Alternative exercise | Total or normalweight women |
| *Sun YC et al (2010) (19)* | Alternative exercise | Total or normalweight women |
| *Bacchi M et al (2018) (20)* | Combined exercise | Total or normalweight women |
| *Backhausen MG et al (2017) (21)* | Combined exercise | Total or normalweight women |
| *Barakat R et al (2011) (22)* | Combined exercise | Total or normalweight women |
| *Barakat R et al (2012) (23)* | Combined exercise | Total or normalweight women |
| *Barakat R et al (2013) (24)* | Combined exercise | Total or normalweight women |
| *Barakat R et al (2018) (25)* | Combined exercise | Total or normalweight women |
| *Barakat R et al (2019) (26)* | Combined exercise | Total or normalweight women |
| *Brik M et al (2018) (27)* | Combined exercise | Total or normalweight women |
| *Cordero Y et al (2012) (28)* | Combined exercise | Total or normalweight women |
| *Cordero Y et al (2015) (29)* | Combined exercise | Total or normalweight women |
| *da Silva SG et al (2017) (30)* | Combined exercise | Total or normalweight women |
| *Fernández-Buhigas I et al (2020) (31)* | Combined exercise | Total or normalweight women |
| *Haakstad LA et al (2011) (32)* | Combined exercise | Total or normalweight women |
| *Peláez M et al (2019) (33)* | Combined exercise | Total or normalweight women |
| *Perales M et al (2016) (34)* | Combined exercise | Total or normalweight women |
| *Price BB et al (2012) (35)* | Combined exercise | Total or normalweight women |
| *Ramírez-Vélez R et al (2017)* *(36)* | Combined exercise | Total or normalweight women |
| *Rodríguez-Blanque R et al (2017) (37)* | Combined exercise | Total or normalweight women |
| *Stafne SN et al (2012) (38)* | Combined exercise | Total or normalweight women |
| *Abd El Hameed AA et al (2011) (39)* | Metformin | Polycystic ovary syndrome |
| *Abd El Fattah EA et al (2016) (40)* | Metformin | Not outcomes of interest |
| *Begum MR et al (2009) (41)* | Metformin | Polycystic ovary syndrome |
| *Glueck CJ et al (2002) (42)* | Metformin | Polycystic ovary syndrome |
| *Jamal A et al (2012) (43)* | Metformin | Polycystic ovary syndrome |
| *Khattab S et al (2011) (44)* | Metformin | Polycystic ovary syndrome |
| *Løvvik TS et al (2019) (45)* | Metformin | Polycystic ovary syndrome |
| *Valdés E et al (2018) (46)* | Metformin | Other risk factors |
| *Vanky E et al (2004) (47)* | Metformin | Polycystic ovary syndrome |
| *Vanky E et al (2010) (48)* | Metformin | Polycystic ovary syndrome |

Supplementary references

1. Cavalcante SR, Cecatti JG, Pereira RI, Baciuk EP, Bernardo AL, Silveira C. Water aerobics II: maternal body composition and perinatal outcomes after a program for low risk pregnant women. Reprod Health. 2009 Jan;6(1):1.
2. Carrascosa MDC, Navas A, Artigues C, Ortas S, Portells E, Soler A, et al. Effect of aerobic water exercise during pregnancy on epidural use and pain: A  multi-centre, randomised, controlled trial. Midwifery. 2021 Jul;103:103105.
3. Clapp JF 3rd, Kim H, Burciu B, Lopez B,. Beginning regular exercise in early pregnancy: effect on fetoplacental growth. Am J Obstet Gynecol [Internet]. 2000 Dec;183(6):1484–8. Available from: <https://www.scopus.com/inward/record.uri?eid=2-s2.0-0034525288&doi=10.1067%2Fmob.2000.107096&partnerID=40&md5=f78543d573b40b807ab84030a607c220>
4. de Oliveria Melo AS, Silva JLP, Tavares JS, Barros VO, Leite DFB, Amorim MMR. Effect of a physical exercise program during pregnancy on uteroplacental and fetal blood flow and fetal growth: A randomized controlled trial. Obstet Gynecol [Internet]. 2012 [cited 2021 Mar 20];120(2):302–10. Available from: <https://pubmed.ncbi.nlm.nih.gov/22825089/>
5. Ghodsi Z, Asltoghiri M. Effects of aerobic exercise training on maternal and neonatal outcome: a randomized controlled trial on pregnant women in Iran. J Pak Med Assoc. 2014 Sep;64(9):1053‐1056.
6. Guelfi KJ, Ong MJ, Crisp NA, Fournier PA, Wallman KE, Grove JR, et al. Regular Exercise to Prevent the Recurrence of Gestational Diabetes Mellitus: a Randomized Controlled Trial. Obstet Gynecol [Internet]. 2016 Oct 1 [cited 2020 Mar 18];128(4):819‐827. Available from: <http://www.ncbi.nlm.nih.gov/pubmed/27607876>
7. Hopkins SA, Baldi JC, Cutfield WS, McCowan L, Hofman PL. Effects of exercise training on maternal hormonal changes in pregnancy. Clin Endocrinol (Oxf). 2011 Apr;74(4):495‐500.
8. Kasawara KT, Burgos CSG, do Nascimento SL, Ferreira NO, Surita FG, Pinto E Silva JL. Maternal and perinatal outcomes of exercise in pregnant women with chronic hypertension and/or previous preeclampsia: a randomized controlled trial. ISRN Obstet Gynecol. 2013;2013:857047.
9. Khoram S, Loripoor M, Pirhadi M, Beigi M. The effect of walking on pregnancy blood pressure disorders in women susceptible  to pregnancy hypertension: A randomized clinical trial. J Educ Health Promot. 2019;8:95.
10. Kihlstrand M, Stenman B, Nilsson S, Axelsson O. Water-gymnastics reduced the intensity of back/low back pain in pregnant women. Acta Obstet Gynecol Scand. 1999 Mar;78(3):180–5.
11. Ko CW, Napolitano PG, Lee SP, Schulte SD, Ciol MA, Beresford SAA. Physical activity, maternal metabolic measures, and the incidence of gallbladder sludge or stones during pregnancy: a randomized trial. Am J Perinatol [Internet]. 2014 Jan [cited 2020 Mar 18];31(1):39‐48. Available from: <http://www.ncbi.nlm.nih.gov/pubmed/23456902>
12. Labonte-Lemoyne E, Curnier D, Ellemberg D. Exercise during pregnancy enhances cerebral maturation in the newborn: a randomized controlled trial. J Clin Exp Neuropsychol. 2017 May;39(4):347‐354.
13. McDonald SM, Newton E, Strickland D, Isler C, Haven K, Kelley G, et al. Influence of Prenatal Aerobic Exercise on Fetal Morphometry. Matern Child Health J. 2020 Nov;24(11):1367–75.
14. Marquez-Sterling S, Perry AC, Kaplan TA, Halberstein RA, Signorile JF. Physical and psychological changes with vigorous exercise in sedentary primigravidae. Med Sci Sports Exerc. 2000 Jan;32(1):58–62.
15. Sedaghati P, Ziaee V, Ardjmand A. The effect of an ergometric training program on pregnants’ weight gain and low back pain. Gazz Med Ital. 2007;166:209–13.
16. Taniguchi C, Sato C. Home-based walking during pregnancy affects mood and birth outcomes among sedentary women: A randomized controlled trial. Int J Nurs Pract. 2016 Oct;22(5):420–6.
17. Tomić V, Sporiš G, Tomić J, Milanović Z, Zigmundovac-Klaić D, Pantelić S. The effect of maternal exercise during pregnancy on abnormal fetal growth. Croat Med J [Internet]. 2013;54(4):362–8. Available from: <https://www.scopus.com/inward/record.uri?eid=2-s2.0-84884936695&doi=10.3325%2Fcmj.2013.54.362&partnerID=40&md5=d4d4a9e43f436bbbd591e974b1f3f228>
18. Rakhshani A, Nagarathna R, Mhaskar R, Mhaskar A, Thomas A, Gunasheela S, et al. The effects of yoga in prevention of pregnancy complications in high-risk pregnancies: a randomized controlled trial. Prev Med (Baltim) [Internet]. 2012 Oct [cited 2020 Mar 18];55(4):333–40. Available from: <http://www.ncbi.nlm.nih.gov/pubmed/22884667>
19. Sun Y-C, Hung Y-C, Chang Y, Kuo S-C. Effects of a prenatal yoga programme on the discomforts of pregnancy and maternal childbirth self-efficacy in Taiwan. Midwifery. 2010 Dec;26(6):e31-6.
20. Bacchi M, Mottola MF, Perales M, Refoyo I, Barakat R. Aquatic Activities During Pregnancy Prevent Excessive Maternal Weight Gain and Preserve Birth Weight: A Randomized Clinical Trial. Am J Heal Promot. 2018 Mar;32(3):729‐735.
21. Backhausen MG, Tabor A, Albert H, Rosthoj S, Damm P, Hegaard HK, et al. The effects of an unsupervised water exercise program on low back pain and sick leave among healthy pregnant women - A randomised controlled trial. PLoS One [Internet]. 2017 Sep 1 [cited 2020 Mar 18];12(9) (no pagination):e0182114. Available from: <http://www.ncbi.nlm.nih.gov/pubmed/28877165>
22. Barakat R, Pelaez M, Montejo R, Luaces M, Zakynthinaki M. Exercise during pregnancy improves maternal health perception: a randomized controlled trial. Am J Obstet Gynecol. 2011 May;204(5):402.e1‐7.
23. Barakat R, Pelaez M, Lopez C, Montejo R, Coteron J. Exercise during pregnancy reduces the rate of cesarean and instrumental deliveries: results of a randomized controlled trial. J Matern neonatal Med. 2012 Nov;25(11):2372–6.
24. Barakat R, Pelaez M, Lopez C, Lucia A, Ruiz JR. Exercise during pregnancy and gestational diabetes-related adverse effects: a randomised controlled trial. Br J Sports Med [Internet]. 2013 Jul [cited 2020 Mar 18];47(10):630–6. Available from: <http://www.ncbi.nlm.nih.gov/pubmed/23365418>
25. Barakat R, Vargas M, Brik M, Fernandez I, Gil J, Coteron J, et al. Does Exercise During Pregnancy Affect Placental Weight?: a Randomized Clinical Trial. Eval Health Prof. 2018 Sep;41(3):400–14.
26. Barakat R, Refoyo I, Coteron J, Franco E. Exercise during pregnancy has a preventative effect on excessive maternal weight gain and gestational diabetes. A randomized controlled trial. Brazilian J Phys Ther [Internet]. 2019 Mar 1 [cited 2020 Mar 18];23(2):148‐155. Available from: <http://www.ncbi.nlm.nih.gov/pubmed/30470666>
27. Brik M, Fernandez-Buhigas I, Martin-Arias A, Vargas-Terrones M, Barakat R, Santacruz B. Does exercise during pregnancy impact on maternal weight gain and fetal cardiac function? A randomized controlled trial. Ultrasound Obstet Gynecol. 2019 May;53(5):583–9.
28. Cordero Y, Peláez M, De Miguel M, Perales M, Barakat R. Can moderate physical exercise during pregnancy act as a factor in preventing gestational diabetes? [¿Puede el ejercicio físico moderado durante el embarazo actuar como un factor de prevención de la diabetes gestacional?]. RICYDE Rev Int Ciencias del Deport [Internet]. 2012;8(27):3–19. Available from: <https://www.scopus.com/inward/record.uri?eid=2-s2.0-84855489307&doi=10.5232%2Fricyde2012.02701&partnerID=40&md5=e5ec7342cc69caaa1dfd07e7c4344b0e>
29. Cordero Y, Mottola MF, Vargas J, Blanco M, Barakat R. Exercise Is Associated with a Reduction in Gestational Diabetes Mellitus. Med Sci Sports Exerc. 2015 Jul 4;47(7):1328‐1333.
30. da Silva SG, Hallal PC, Domingues MR, Bertoldi ADAD, Silveira MF da MF da, Bassani D, et al. A randomized controlled trial of exercise during pregnancy on maternal and neonatal outcomes: results from the PAMELA study. Int J Behav Nutr Phys Act [Internet]. 2017 Dec 22 [cited 2020 Mar 18];14(1):175. Available from: <http://www.ncbi.nlm.nih.gov/pubmed/29273044>
31. Fernández-Buhigas I, Brik M, Martin-Arias A, Vargas-Terrones M, Varillas D, Barakat R, et al. Maternal physiological changes at rest induced by exercise during pregnancy: a randomized controlled trial. Physiol Behav. 2020 Jun;220:112863.
32. Haakstad LAH, Bo K. Effect of regular exercise on prevention of excessive weight gain in pregnancy: a randomised controlled trial. Eur J Contracept Reprod Health Care. 2011 Apr;16(2):116–25.
33. Pelaez M, Gonzalez-Cerron S, Montejo R, Barakat R. Protective Effect of Exercise in Pregnant Women Including Those Who Exceed Weight Gain Recommendations: A Randomized Controlled Trial. Mayo Clin Proc [Internet]. 2019 Oct 1 [cited 2020 Mar 18];94(10):1951–9. Available from: <http://www.ncbi.nlm.nih.gov/pubmed/31585579>
34. Perales M, Calabria I, Lopez C, Franco E, Coteron J, Barakat R. Regular Exercise Throughout Pregnancy Is Associated With a Shorter First Stage of Labor. Am J Health Promot. 2016;30(3):149–54.
35. Price BB, Amini SB, Kappeler K. Exercise in pregnancy: effect on fitness and obstetric outcomes-a randomized trial. Med Sci Sports Exerc [Internet]. 2012 Dec [cited 2020 Mar 18];44(12):2263‐2269. Available from: <http://www.ncbi.nlm.nih.gov/pubmed/22843114>
36. Ramírez-Vélez R, Lobelo F, Aguilar-de Plata AC, Izquierdo M, García-Hermoso A. Exercise during pregnancy on maternal lipids: A secondary analysis of randomized controlled trial. BMC Pregnancy Childbirth [Internet]. 2017 Nov;17(1):396. Available from: <https://www.scopus.com/inward/record.uri?eid=2-s2.0-85035149904&doi=10.1186%2Fs12884-017-1571-6&partnerID=40&md5=8578115b4f856028021265bda988cd8d>
37. Rodríguez-Blanque R, Sánchez-García JC, Sánchez-López AM, Mur-Villar N, Fernández-Castillo R, Aguilar Cordero MJ. [Influence of physical exercise during pregnancy on newborn weight: a randomized  clinical trial]. Nutr Hosp. 2017 Jul;34(4):834–40.
38. Stafne SN, Salvesen KÅ, Romundstad PR, Eggebø TM, Carlsen SM, Mørkved S. Regular exercise during pregnancy to prevent gestational diabetes: A randomized controlled trial. Obstet Gynecol [Internet]. 2012 Jan [cited 2020 Mar 18];119(1):29–36. Available from: <http://www.ncbi.nlm.nih.gov/pubmed/22183208>
39. Abd El Hameed AA, Shreif HE, Mowafy HE. The role of continuing metformin therapy during pregnancy in the reduction of gestational diabetes and improving pregnancy outcomes in women with polycystic ovary syndrome. Middle East Fertil Soc J. 2011 Sep;16(3):204–8.
40. Fattah E. Can metformin limit weight gain in the obese with pregnancy? Int J Reprod Contraception, Obstet Gynecol. 2016 Feb 23;5(3):818–25.
41. Begum MR, Khanam NN, Quadir E, Ferdous J, Begum MS, Khan F, et al. Prevention of gestational diabetes mellitus by continuing metformin therapy throughout pregnancy in women with polycystic ovary syndrome. J Obstet Gynaecol Res. 2009 Apr;35(2):282–6.
42. Glueck CJ, Wang P, Kobayashi S, Phillips H, Sieve-Smith L. Metformin therapy throughout pregnancy reduces the development of gestational diabetes in women with polycystic ovary syndrome. Fertil Steril [Internet]. 2002 Mar [cited 2020 Mar 18];77(3):520–5. Available from: <http://www.ncbi.nlm.nih.gov/pubmed/11872206>
43. Jamal A, Milani F, Al-Yasin A. Evaluation of the effect of metformin and aspirin on utero placental circulation of pregnant women with PCOS. Iran J Reprod Med [Internet]. 2012;10(3):265–70. Available from: <https://www.scopus.com/inward/record.uri?eid=2-s2.0-84865618588&partnerID=40&md5=c7a20374bb6a01b28bd1995268bd6ef1>
44. Khattab S, Mohsen IA, Aboul Foutouh I, Ashmawi HS, Mohsen MN, Van Wely M, et al. Can metformin reduce the incidence of gestational diabetes mellitus in pregnant women with polycystic ovary syndrome? Prospective cohort study. Gynecol Endocrinol [Internet]. 2011 Oct [cited 2020 Mar 18];27(10):789–93. Available from: <http://www.ncbi.nlm.nih.gov/pubmed/21247239>
45. Løvvik TS, Carlsen SM, Salvesen Ø, Steffensen B, Bixo M, Gómez-Real F, et al. Use of metformin to treat pregnant women with polycystic ovary syndrome (PregMet2): a randomised, double-blind, placebo-controlled trial. Lancet Diabetes Endocrinol. 2019 Apr 1;7(4):256–66.
46. Valdés E, Sepúlveda-Martínez A, Candia P, Abusada N, Orellana R, Manukian BBB, et al. Metformin as a prophylactic treatment of gestational diabetes in pregnant patients with pregestational insulin resistance: a randomized study. J Obstet Gynaecol Res [Internet]. 2018 Jan 1 [cited 2020 Mar 18];44(1):81‐86. Available from: <http://www.ncbi.nlm.nih.gov/pubmed/29094444>
47. Vanky E, Salvesen KA, Heimstad R, Fougner KJ, Romundstad P, Carlsen SM, et al. Metformin reduces pregnancy complications without affecting androgen levels in pregnant polycystic ovary syndrome women: results of a randomized study. Hum Reprod [Internet]. 2004 Aug [cited 2020 Mar 18];19(8):1734–40. Available from: <http://www.ncbi.nlm.nih.gov/pubmed/15178665>
48. Vanky E, Stridsklev S, Heimstad R, Romundstad P, Skogøy K, Kleggetveit O, et al. Metformin Versus placebo from first trimester to delivery in polycystic ovary syndrome: A randomized, controlled multicenter study. J Clin Endocrinol Metab [Internet]. 2010 Dec [cited 2020 Mar 18];95(12):E448-55. Available from: <http://www.ncbi.nlm.nih.gov/pubmed/20926533>

**Table S3.** Characteristics of the interventions.

| Reference | Intervention | Control |
| --- | --- | --- |
| A. Exercise | | |
| *Barakat R et al (2016)* | Duration: 28 weeks (9-11 week until 38-39 week).  Frequency: 3 times/week.  Exercises: 50-55 min. Warm up/cool-down, walking, aerobic exercise, aerobic dance, resistance exercises (2 kg weights, low resistance bands), flexibility, relaxation stretching, pelvic floor muscle training.  Type: Combined exercise.  Intensity: Moderate (<70% of predicted maximum heart rate, Borg: 12-14). | The women received general advice from their healthcare provider about the positive effects of physical activity.  They had the same visits with health care providers as the intervention group.  A questionnaire was conducted once a quarter. Those women who exercised continuously were excluded from the analysis. |
| *Bisson M et al (2015)* | Duration: 12 weeks (15 week until 27 week).  Frequency: 3 times/week.  Exercises: 60 min. Warm up/cool-down, stationary ergocycle, treadmill walk, muscular work-out.  Type: Combined exercise.  Intensity: Moderate (70% of peak heart rate, modified Borg: 3-5). | The women were asked to continue with their usual activities, without restricting physical activity.  Both groups received a pamphlet of the benefits of physical activity and the exercises they could do. |
| *Daly N et al (2017)* | Duration: 22 weeks (>17 week until delivery).  Frequency: 3 times week.  Exercises: 50-60 min. Warm up/cool-down, resistance or weight exercises, aerobic exercises, pelvic floor muscle training.  Type: Combined exercise.  Intensity: NA. | The women received written information about the exercise.  Both groups received a pamphlet with information about healthy eating. |
| *Garnæs KK et al (2016)* | Duration: 25 weeks (12-18 week until 39 week).  Frequency: 4 times/week.  Exercises: 60 min. Walking/jogging, resistance training, pelvic floor muscle training.  Type: Combined exercise.  Intensity: Moderate (~80% of maximal capacity, Borg: 12-15). | Information on healthy eating and lifestyles was provided.  The pregnant women were asked to continue with their usual activities and were not discouraged from exercising. |
| *Kong K et al (2014)* | Duration: 20 weeks (15 week until 35 week).  Frequency: 5 times/week.  Exercises: 30 min. Walk (Treadmills).  Type: Aerobic exercise.  Intensity: Moderate (cadence ≥80 steps per minute). | The women received no recommendations for physical activity, but they were not discouraged from doing so. |
| *Nascimento SL et al (2011)* | Duration: ~18 weeks (~20 week until delivery).  Frequency: 5 times/week.  Exercises: 40 min. General stretching, strength exercises, relaxation, walking.  Type: Combined exercise.  Intensity: Moderate (<140 beats per minute of heart rate). | The women received no recommendations for physical activity.  Both groups received standardized nutritional counselling. |
| *Oostdam N et al (2012)* | Duration: 24 weeks (15 week until 39 week).  Frequency: 2 times/week.  Exercises: 60 min. Warm up/cool-down, aerobic and strength exercises.  Type: Combined exercise.  Intensity: Moderate (Borg: 12). | The women did not receive any exercise program, but received the usual care, which is the same as women of a healthy weight. |
| *Ruiz JR et al (2013)* | Duration: 30 weeks (9 week until 39 week).  Frequency: 3 times week.  Exercises: 50-55 min. Warm-up/cool-down, waking, stretching, aerobic exercises, aerobic dance, resistance exercises, barbells (3kg), low-medium elastic bands.  Type: Combined exercise.  Intensity: Light-Moderate (<60% age predicted maximum heart rate, Borg: 10-12). | The women received standard care. Counselling on nutrition and physical activity was included, and exercise was not discouraged. |
| *Santos IA et al (2005)* | Duration: 12 weeks.  Frequency: 3 times/week.  Exercises: 60 min. Warm-up/cool-down, aerobic exercises (walking, pedalling a bicycle ergometer, and aerobic gymnastics), stretching, resistance exercises (dumbbells up to 1 kg, rods, tennis balls, body weight resistance exercises such as squats and lunges).  Type: Combined.  Intensity: Light-Moderate (50-60% of the maximum predicted heart rate, <140 beats per minute). | Once-weekly sessions including relaxation and focus group discussions concerning maternity, without encouraging or discouraging from exercising. |
| *Seneviratne SN et al (2016)* | Duration: 15 weeks (20 week until 35 week).  Frequency: 3-5 times/week.  Exercises: 15-30 min. Stationary bicycle.  Type: Aerobic exercise.  Intensity: Moderate (40–59% VO_2_ reserve). | The women did not receive the exercise program. |
| *Wang C et al (2017)* | Duration: 25 weeks (11 week until 36 week).  Frequency: 3 times/week.  Exercises: 45-60 min. Interval stationary cycling (Intensity intervals between moderate level, 65-75% of the age-predicted HRmax, Borg 12-14, and vigorous level, 75-85% of the age-predicted maximum heart rate, Borg 15-16).  Type: Aerobic exercise.  Intensity: Moderate-Vigorous. | The women continued with their usual activities, and physical exercise was not discouraged. |
| B. Metformin | | |
| *Brink HS et al (2018)* | Duration: 25 weeks (14 week until 39 week).  Dose: 1000 mg/day. | Women in both groups followed a 2000 Kcal/day diet. |
| *Chiswick C et al (2015)* | Duration: 25 weeks (12-16 week until 39 week).  Dose: 2500 mg/day. | Placebo was administered. |
| *Nascimento IB et al (2020)* | Duration: >20 weeks (<20 week until delivery).  Dose: 1000 mg/day. | Women in both groups received the usual health care.  Both groups received nutritional and physical activity recommendations. |
| *Syngelaki A et al (2016)* | Duration: 25 weeks (12-18 week until delivery).  Dose: 3000 mg/day. | Placebo was administered.  Both groups received nutritional and physical activity recommendations. |

NA: Not available

**Table S4.** Results for direct pairwise comparisons and network meta-analysis.

| 1. Caesarean section | | | | |
| --- | --- | --- | --- | --- |
|  | **Control** | **Aerobic** | **Combined** | **Metformin** |
| **Control** |  | 1.04  (0.70, 1.52) | 0.99  (0.80, 1.21) | 0.79*  (0.63, 0.99) |
| **Aerobic** | 1.00  (0.56, 1.77) |  | - | - |
| **Combined** | 1.02  (0.67, 1.57) | 1.03  (0.50, 2.10) |  | - |
| **Metformin** | 0.66*  (0.46, 0.95) | 0.66  (0.34, 1.31) | 0.65  (0.37, 1.13) |  |
| 1. Preterm delivery | | | | |
| **Control** |  | 0.76  (0.26, 2.24) | 0.99  (0.50, 1.96) | 0.89  (0.55, 1.46) |
| **Aerobic** | 0.77  (0.23, 2.58) |  | - | - |
| **Combined** | 1.06  (0.45, 2.50) | 1.39  (0.32, 6.06) |  | - |
| **Metformin** | 0.88  (0.48, 1.61) | 1.15  (0.29, 4.46) | 0.82  (0.28, 2.39) |  |
| 1. Macrosomia | | | | |
| **Control** |  | 0.94  (0.49, 1.80) | 0.46  (0.14, 1.52) | 0.97  (0.28, 3.28) |
| **Aerobic** | 0.93  (0.39, 2.26) |  | - | - |
| **Combined** | 0.37*  (0.14, 0.95) | 0.39  (0.11, 1.44) |  | - |
| **Metformin** | 0.96  (0.19, 4.84) | 1.03  (0.16, 6.49) | 2.62  (0.40, 17.06) |  |
| 1. Birthweight | | | | |
| **Control** |  | -88.05  (-224.15, 48.06) | 2.97  (-71.10, 77.04) | -64.53  (-246.23, 117.16) |
| **Aerobic** | -96.66*  (-192.45, -0.88) |  | - | - |
| **Combined** | 2.62  (-71.33, 76.56) | 99.28  (-21.73, 220.29) |  | - |
| **Metformin** | -33.08  (-137.48, 71.33) | 63.58  (-78.10, 205.27) | -35.69  (-163.63, 92.24) |  |

Caesarean section, preterm delivery and macrosomia were measured as risk ratio (95% CI). Birthweight was measured as mean difference (95% CI). Upper diagonal: standard meta-analysis. Lower diagonal: network meta-analysis estimates. *Statistical significance.

**Table S5.** Grades of Recommendation, Assessment, Development, and Evaluation.

| Certainty assessment | | | | | | | Impact | Certainty |
| --- | --- | --- | --- | --- | --- | --- | --- | --- |
| № of studies | **Study design** | **Risk of bias** | **Inconsistency** | **Indirectness** | **Imprecision** | **Other considerations** |  |  |
| A. Caesarean delivery | | | | | | | | |
| Aerobic exercise | | | | | | | | |
| 3 | RCT | very serious | serious | serious | serious | none | Meta-analysis: RR = 1.04 (95% CI: 0.70, 1.52)  NMA: RR = 1.00 (95% CI: 0.56, 1.77) | VERY LOW |
| Combined exercise | | | | | | | | |
| 6 | RCT | very serious | not serious | serious | serious | none | Meta-analysis: RR = 0.99 (95% CI: 0.80, 1.21)  NMA: RR = 1.02 (95% CI: 0.67, 1.57) | VERY LOW |
| Metformin | | | | | | | | |
| 4 | RCT | very serious | not serious | serious | not serious | strong association | Meta-analysis: RR = 0.79 (95%CI: 0.63, 0.99)  NMA: RR = 0.66 (95%CI: 0.46, 0.95) | LOW |
| B. Preterm birth | | | | | | | | |
| Aerobic exercise | | | | | | | | |
| 3 | RCT | very serious | not serious | serious | very serious | none | Meta-analysis: RR = 0.76 (95% CI: 0.26, 2.24)  NMA: RR = 0.77 (95% CI: 0.23, 2.58) | VERY LOW |
| Combined exercise | | | | | | | | |
| 4 | RCT | very serious | not serious | serious | serious | none | Meta-analysis: RR = 0.99 (95% CI: 0.50, 1.96)  NMA: RR = 1.06 (95% CI: 0.45, 2.50) | VERY LOW |
| Metformin | | | | | | | | |
| 4 | RCT | very serious | not serious | serious | serious | none | Meta-analysis: RR = 0.89 (95% CI: 0.55, 1.46)  NMA: RR = 0.88 (95% CI: 0.48 1.61) | VERY LOW |
| C. Macrosomia | | | | | | | | |
| Aerobic exercise | | | | | | | | |
| 3 | RCT | very serious | not serious | serious | serious | none | Meta-analysis: RR = 0.94 (95% CI: 0.49, 1.80)  NMA: RR = 0.93 (95% CI: 0.39, 2.26) | VERY LOW |
| Combined exercise | | | | | | | | |
| 4 | RCT | very serious | serious | serious | not serious | very strong association | Meta-analysis: RR = 0.46 (95% CI: 0.14, 1.52)  NMA: RR = 0.37 (95% CI: 0.14, 0.95) | LOW |
| Metformin | | | | | | | | |
| 1 | RCT | serious | not serious | serious | very serious | none | Meta-analysis: RR = 0.97 (95% CI: 0.28, 3.28)  NMA: RR = 0.96 (95% CI: 0.19, 4.84) | VERY LOW |
| D. Birth weight | | | | | | | | |
| Aerobic exercise | | | | | | | | |
| 3 | RCT | very serious | not serious | serious | not serious | none | Meta-analysis = -88.05 g. (95% CI: -224.15, 48.06)  NMA = -96.66 g. (95% CI: -192.45, -0.88) | VERY LOW |
| Combined exercise | | | | | | | | |
| 7 | RCT | very serious | not serious | serious | not serious | none | Meta-analysis = 2.97 g. (95% CI: -71.10, 77.04)  NMA = 2.62 g. (95% CI: -71.33, 76.56) | VERY LOW |
| Metformin | | | | | | | | |
| 2 | RCT | very serious | not serious | serious | not serious | none | Meta-analysis = -64.53 g. (95% CI: -246.23, 117.16)  NMA = -33.08 g. (95% CI: -137.48, 71.33) | VERY LOW |

RCT: Randomized clinical trial, NMA: Network meta-analysis.

**Table S6.** Transitivity analysis.

**A.** Caesarean section

| Reference | Age | | | | BMI | | | |
| --- | --- | --- | --- | --- | --- | --- | --- | --- |
|  | **Intervention** | | **Control** | | **Intervention** | | **Control** | |
|  | **** | **SD** | **** | **SD** | **** | **SD** | **** | **SD** |
| Physical exercise | | | | | | | | |
| *Bisson M et al (2015)* | 30.5 | 3.7 | 31.0 | 4.0 | 34.6 | 5.4 | 33.9 | 4.5 |
| *Daly N et al (2017)* | 30.0 | 5.1 | 29.4 | 4.8 | 34.7 | 4.6 | 34.7 | 5.1 |
| *Garnæs KK et al (2016)* | 31.3 | 3.8 | 31.4 | 4.7 | 33.9 | 3.8 | 35.1 | 4.6 |
| *Kong K et al (2014)-1* | 26.2 | 2.6 | 27.3 | 3.6 | 26.5 | 1.2 | 27.4 | 1.4 |
| *Kong K et al (2014)-2* | 28.6 | 5.3 | 25.7 | 4.0 | 34.7 | 4.6 | 34.2 | 3.6 |
| *Nascimento SL et al (2011)* | 29.7 | 6.8 | 30.9 | 5.9 | 34.8 | 6.6 | 36.4 | 6.9 |
| *Oostdam N et al (2012)* | 30.8 | 5.2 | 30.1 | 4.5 | 33.0 | 3.7 | 33.9 | 5.6 |
| *Ruiz JR et al (2013)* | - | - | - | - | - | - | - | - |
| *Seneviratne SN et al (2016)* | - | - | - | - | 32.1 | 4.4 | 34.1 | 5.9 |
| *Wang C et al (2017)* | 32.1 | 4.6 | 32.5 | 4.9 | 26.8 | 2.7 | 26.8 | 2.8 |
| Pooled | **30.1** | **1.8** | **30.0** | **1.9** | **32.3** | **4.0** | **32.9** | **4.4** |
| Metformin | | | | | | | | |
| *Brink HS et al (2018)* | 29.3 | 5.2 | 30.7 | 5.2 | 31.3 | 5.8 | 30.0 | 5.5 |
| *Chiswick C et al (2015)* | 28.7 | 5.8 | 28.9 | 5.1 | 37.8 | 4.7 | 37.5 | 5.5 |
| *Nascimento IB et al (2020)* | 28.6 | 6.2 | 29.6 | 6.1 | 37.5 | 4.6 | 37.2 | 5.7 |
| *Syngelaki A et al (2016)* | - | - | - | - | - | - | - | - |
| Pooled | **28.7** | **0.5** | **29.4** | **0.7** | **36.1** | **1.7** | **35.2** | **2.4** |

*Statistically significant difference

**B.** Preterm birth

| Reference | Age | | | | BMI | | | |
| --- | --- | --- | --- | --- | --- | --- | --- | --- |
|  | **Intervention** | | **Control** | | **Intervention** | | **Control** | |
|  | **** | **SD** | **** | **SD** | **** | **SD** | **** | **SD** |
| Physical exercise | | | | | | | | |
| *Barakat R et al (2016)-1* | - | - | - | - | - | - | - | - |
| *Barakat R et al (2016)-2* | - | - | - | - | - | - | - | - |
| *Daly N et al (2017)* | 30.0 | 5.1 | 29.4 | 4.8 | 34.7 | 4.6 | 34.7 | 5.1 |
| *Garnæs KK et al (2016)* | 31.3 | 3.8 | 31.4 | 4.7 | 33.9 | 3.8 | 35.1 | 4.6 |
| *Kong K et al (2014)-1* | 26.2 | 2.6 | 27.3 | 3.6 | 26.5 | 1.2 | 27.4 | 1.4 |
| *Kong K et al (2014)-2* | 28.6 | 5.3 | 25.7 | 4.0 | 34.7 | 4.6 | 34.2 | 3.6 |
| *Ruiz JR et al (2013)* | - | - | - | - | - | - | - | - |
| *Santos IA et al (2005)* | 26.0 | 3.4 | 28.6 | 5.9 | 28.0 | 2.1 | 27.5 | 2.1 |
| *Seneviratne SN et al (2016)* | - | - | - | - | 32.1 | 4.4 | 34.1 | 5.9 |
| *Wang C et al (2017)* | 32.1 | 4.6 | 32.5 | 4.9 | 26.8 | 2.7 | 26.8 | 2.8 |
| Pooled | **29.1** | **3.1** | **29.3** | **2.5** | **30.8*** | **3.1** | **31.3** | **3.3** |
| Metformin | | | | | | | | |
| *Brink HS et al (2018)* | 29.3 | 5.2 | 30.7 | 5.2 | 31.3 | 5.8 | 30.0 | 5.5 |
| *Chiswick C et al (2015)* | 28.7 | 5.8 | 28.9 | 5.1 | 37.8 | 4.7 | 37.5 | 5.5 |
| *Nascimento IB et al (2020)* | 28.6 | 6.2 | 29.6 | 6.1 | 37.5 | 4.6 | 37.2 | 5.7 |
| *Syngelaki A et al (2016)* | - | - | - | - | - | - | - | - |
| Pooled | **28.7** | **0.5** | **29.4** | **0.7** | **36.1*** | **1.7** | **35.2** | **2.4** |

*Statistically significant difference, : mean; SD: standard deviation.

**C.** Macrosomia

| Reference | Age | | | | BMI | | | |
| --- | --- | --- | --- | --- | --- | --- | --- | --- |
|  | **Intervention** | | **Control** | | **Intervention** | | **Control** | |
|  | **** | **SD** | **** | **SD** | **** | **SD** | **** | **SD** |
| Physical exercise | | | | | | | | |
| *Barakat R et al (2016)-1* | - | - | - | - | - | - | - | - |
| *Barakat R et al (2016)-2* | - | - | - | - | - | - | - | - |
| *Daly N et al (2017)* | 30.0 | 5.1 | 29.4 | 4.8 | 34.7 | 4.6 | 34.7 | 5.1 |
| *Garnæs KK et al (2016)* | 31.3 | 3.8 | 31.4 | 4.7 | 33.9 | 3.8 | 35.1 | 4.6 |
| *KONG KAIL et al (2014)-1* | 26.2 | 2.6 | 27.3 | 3.6 | 26.5 | 1.2 | 27.4 | 1.4 |
| *KONG KAIL et al (2014)-2* | 28.6 | 5.3 | 25.7 | 4.0 | 34.7 | 4.6 | 34.2 | 3.6 |
| *Ruiz JR et al (2013)* | - | - | - | - | - | - | - | - |
| *Seneviratne SN et al (2016)* | - | - | - | - | 32.1 | 4.4 | 34.1 | 5.9 |
| *Wang C et al (2017)* | 32.1 | 4.6 | 32.5 | 4.9 | 26.8 | 2.7 | 26.8 | 2.8 |
| Pooled | **29.8** | **2.3** | **29.5** | **2.5** | **31.3** | **3.9** | **31.9** | **4.2** |
| Metformin | | | | | | | | |
| *Syngelaki A et al (2016)* | - | - | - | - | - | - | - | - |
| Pooled | **-** | **-** | **-** | **-** | **-** | **-** | **-** | **-** |

*Statistically significant difference

**D.** Birthweight

| Reference | Age | | | | BMI | | | |
| --- | --- | --- | --- | --- | --- | --- | --- | --- |
|  | **Intervention** | | **Control** | | **Intervention** | | **Control** | |
|  | **** | **SD** | **** | **SD** | **** | **SD** | **** | **SD** |
| Physical exercise | | | | | | | | |
| *Bisson M et al (2015)* | 30.5 | 3.7 | 31.0 | 4.0 | 34.6 | 5.4 | 33.9 | 4.5 |
| *Daly N et al (2017)* | 30.0 | 5.1 | 29.4 | 4.8 | 34.7 | 4.6 | 34.7 | 5.1 |
| *Garnæs KK et al (2016)* | 31.3 | 3.8 | 31.4 | 4.7 | 33.9 | 3.8 | 35.1 | 4.6 |
| *KONG KAIL et al (2014)-1* | 26.2 | 2.6 | 27.3 | 3.6 | 26.5 | 1.2 | 27.4 | 1.4 |
| *KONG KAIL et al (2014)-2* | 28.6 | 5.3 | 25.7 | 4.0 | 34.7 | 4.6 | 34.2 | 3.6 |
| *Nascimento SL et al (2011)* | 29.7 | 6.8 | 30.9 | 5.9 | 34.8 | 6.6 | 36.4 | 6.9 |
| *Oostdam N et al (2012)* | 30.8 | 5.2 | 30.1 | 4.5 | 33.0 | 3.7 | 33.9 | 5.6 |
| *Ruiz JR et al (2013)* | - | - | - | - | - | - | - | - |
| *Santos IA et al (2005)* | 26.0 | 3.4 | 28.6 | 5.9 | 28.0 | 2.1 | 27.5 | 2.1 |
| *Seneviratne SN et al (2016)* | - | - | - | - | 32.1 | 4.4 | 34.1 | 5.9 |
| *Wang C et al (2017)* | 32.1 | 4.6 | 32.5 | 4.9 | 26.8 | 2.7 | 26.8 | 2.8 |
| Pooled | **29.5** | **2.6** | **29.9** | **1.9** | **31.8** | **3.5** | **32.3** | **3.7** |
| Metformin | | | | | | | | |
| *Brink HS et al (2018)* | 29.3 | 5.2 | 30.7 | 5.2 | 31.3 | 5.8 | 30.0 | 5.5 |
| *Chiswick C et al (2015)* | 28.7 | 5.8 | 28.9 | 5.1 | 37.8 | 4.7 | 37.5 | 5.5 |
| Pooled | **28.8** | **0.5** | **29.5** | **1.2** | **34.7** | **4.6** | **33.8** | **5.3** |

*Statistically significant difference, : mean; SD: standard deviation.

**Table S7.** Results for direct pairwise comparisons and network meta-analysis among pregnant women with obesity.

| 1. Caesarean section | | | | |
| --- | --- | --- | --- | --- |
|  | **Control** | **Aerobic** | **Combined** | **Metformin** |
| **Control** |  | 1.30  (0.82, 2.06) | 0.99  (0.68, 1.43) | 0.79*  (0.63, 0.99) |
| **Aerobic** | 1.53  (0.59, 3.96) |  | - | - |
| **Combined** | 1.02  (0.55, 1.87) | 0.66  (0.21, 2.06) |  | - |
| **Metformin** | 0.66*  (0.45, 0.97) | 0.43  (0.15, 1.20) | 0.65  (0.32, 1.33) |  |
| 1. Preterm delivery | | | | |
| **Control** |  | 1.70  (0.23, 12.62) | 2.14  (0.61, 7.53) | 0.89  (0.55, 1.46) |
| **Aerobic** | 1.73  (0.21, 14.44) |  | - | - |
| **Combined** | 2.29  (0.58, 9.01) | 1.32  (0.11, 16.47) |  | - |
| **Metformin** | 0.89  (0.53, 1.49) | 0.51  (0.06, 4.53) | 0.39  (0.09, 1.67) |  |
| 1. Macrosomia | | | | |
| **Control** |  | 0.91  (0.30, 2.77) | 0.72  (0.43, 1.22) | 0.97  (0.28, 3.28) |
| **Aerobic** | 0.85  (0.17, 4.27) |  | - | - |
| **Combined** | 0.94  (0.16, 5.50) | 1.11  (0.09, 14.31) |  | - |
| **Metformin** | 0.96  (0.12, 7.47) | 1.14  (0.08, 15.45) | 1.02  (0.07, 15.21) |  |
| 1. Birthweight | | | | |
| **Control** |  | -162.53  (-528.14, 203.08) | 33.87  (-114.92, 182.66) | -64.53  (-246.23, 117.16) |
| **Aerobic** | -133.12  (-410.58, 144.33) |  | - | - |
| **Combined** | 32.84  (-122.19, 187.88) | 165.97  (-149.94, 481.87) |  | - |
| **Metformin** | -67.46  (-266.21, 131.28) | 65.66  (-266.52, 397.84) | -100.31  (-350.12, 149.51) |  |

**Table S8.** Meta-regressions models by body mass index and maternal weight gain.

1. Body mass index and maternal weight gain

| Reference | Body mass index (Kg/m^2^) | Maternal weight gain (Kg) |
| --- | --- | --- |
| *Kong K et al (2014)-1* | 26,75 | 0,59 |
| *Kong K et al (2014)-2* | 34,45 | -0,41 |
| *Seneviratne SN et al (2016)* | 33,1 | -1,2 |
| *Wang C et al (2017)* | 26,79 | -2,09 |
| *Barakat R et al (2016)-1* | NR | NR |
| *Barakat R et al (2016)-2* | NR | NR |
| *Bisson M et al (2015)* | 34,25 | 0,1 |
| *Daly N et al (2017)* | 34,7 | -1,7 |
| *Garnæs KK et al (2017)* | 34,5 | 1,3 |
| *Nascimento SL et al (2011)* | 35,6 | -1,2 |
| *Oostdam N et al (2012)* | 33,45 | 0,6 |
| *Ruiz JR et al (2013)* | NR | -0,5 |
| *Santos IA et al (2005)* | 27,75 | NR |
| *Brink HS et al (2018)* | 30,65 | NR |
| *Chiswick C et al (2015)* | 37,75 | -0,53 |
| *Nascimento IB et al (2020)* | 37,3 | NR |
| *Syngelaki A et al (2016)* | 38,5 | NR |

NR: Not reported

1. Meta-regressions by body mass index or maternal weight gain, and each outcome

| Covariate | Caesarean Section | Preterm birth | Macrosomia | Birthweight |
| --- | --- | --- | --- | --- |
| Body mass index | -0.01 (-0.05, 0.03), n = 13 | 0.01 (-0.09, 0.12), n = 11 | -0.01 (-0.12, 0.10), n = 7 | 7.35 (-10.27, 24.96), n = 12 |
| Maternal weight gain | 0.05 (-0.15, 0.25), n = 11 | 0.29 (-0.44, 1.01), n = 8 | -0.03 (-0.59, 0.52), n = 7 | 37.05 (-28.08, 102.18), n = 11 |

**Table S9.** Meta-regressions models by intervention.

| Covariate | Aerobic exercise | Combined exercise | Metformin |
| --- | --- | --- | --- |
| A. Caesarean section | | | |
| Age | 0.12 (-3.75, 3.99), n=3 | 0.26 (-0.71, 1.23), n=5 | 0.08 (-0.30, 0.46), n=4 |
| Body mass index | 0.10 (-2.43, 2.63), n=3 | -0.07 (-0.63, 0.49), n=5 | 0.03 (-0.23, 0.29), n=4 |
| Intensity | -0.29 (-1.93, 1.34), n=4 | -0.19 (-0.99, 0.61), n=5 | 0.19 (-0.13, 0.53), n=4 |
| Frequency | 0.08 (-1.28, 1.44), n=4 | -0.05 (-0.33, 0.22), n=6 | NA |
| Time/session | -0.00 (-0.02, 0.01), n=4 | 0.01 (-0.03, 0.04), n=6 | NA |
| Initial week | 0.05 (-0.14, 0.23), n=4 | -0.02 (-0.09, 0.04), n=6 | -0.06 (-0.17, 0.05), n=4 |
| Total weeks | -0.04 (-0.21, 0.13), n=4 | 0.02 (-0.04, 0.07), n=6 | 0.07 (-0.05, 0.19), n=4 |
| Total sessions | -0.01 (-0.08, 0.06), n=4 | 0.00 (-0.01, 0.02), n=6 | NA |
| B. Preterm birth | | | |
| Age | 0.03 (-3.31, 3.37), n=3 | 0.19 (-5.71, 6.09), n=3 | -0.25 (-0.88, 0.39), n=4 |
| Body mass index | 0.09 (-3.33, 3.51), n=3 | 0.02 (-0.91, 0.95), n=3 | 0.12 (-0.78, 1.02), n=4 |
| Intensity | -0.54 (-5.35, 4.28), n=4 | -0.73 (-3.80, 2.33), n=5 | -0.09 (-1.72, 1.53), n=4 |
| Frequency | 0.09 (-2.87, 3.05), n=4 | 1.52 (-3.11, 6.15), n=6 | NA |
| Time/session | -0.03 (-0.21, 0.15), n=4 | 0.16 (-0.24, 0.56), n=6 | NA |
| Initial week | 0.12 (-0.54, 0.78), n=4 | 0.09 (-0.22, 0.41), n=6 | 0.03 (-0.55, 0.61), n=4 |
| Total weeks | -0.10 (-0.69, 0.48), n=4 | -0.05 (-0.27, 0.18), n=6 | -0.06 (-0.69, 0.58), n=4 |
| Total sessions | -0.03 (-0.21, 0.15), n=4 | -0.00 (-0.08, 0.07), n=6 | NA |
| C. Macrosomia | | | |
| Age | -0.09 (-3.49, 3.31), n=3 | NC | NC |
| Body mass index | -0.11 (-2.19, 1.97), n=3 | NC | NC |
| Intensity | -0.56 (-4.03, 2.92), n=4 | 1.25 (-6.62, 9.12), n=4 | NC |
| Frequency | 0.15 (-2.13, 2.43), n=4 | 0.49 (-5.38, 6.36), n=5 | NA |
| Time/session | -0.02 (-0.14, 0.09), n=4 | 0.13 (-0.63, 0.89), n=5 | NA |
| Initial week | 0.09 (-0.24, 0.42), n=4 | 0.24 (-0.21, 0.68), n=5 | NC |
| Total weeks | -0.07 (0.38, 0.22), n=4 | -0.31 (-0.83, 0.21), n=5 | NC |
| Total sessions | -0.01 (-0.12, 0.09), n=4 | -0.04 (-0.28, 0.19), n=5 | NA |
| D. Birth weight | | | |
| Age | -5.39 (-1058.21, 1047.43), n=3 | -0.32 (-121.38, 120.75), n=6 | NC |
| Body mass index | -46.20 (-532.89, 440.49), n=3 | 1.51 (-18.42, 21.43), n=6 | NC |
| Intensity | -51.38 (-1034.23, 931.48), n=4 | 82.02 (-167.16, 331.20), n=6 | NC |
| Frequency | 14.65 (-527.89, 557.20), n=4 | -64.19 (-209.38, 80.98), n=7 | NA |
| Time/session | -2.40 (-39.25, 34.44), n=4 | 2.76 (-16.88, 22.40), n=7 | NA |
| Initial week | 10.42 (-115.97, 136.81), n=4 | 5.86 (-18.68, 30.39), n=7 | NC |
| Total weeks | -9.11 (-123.74, 105.53), n=4 | -5.67 (-19.74, 8.39), n=7 | NC |
| Total sessions | -1.81 (-31.28, 27.65), n=4 | -2.79 (-6.91, 1.34), n=7 | NA |

**Figure S1.** Direct pairwise comparisons by outcome.

**B.** Preterm birth


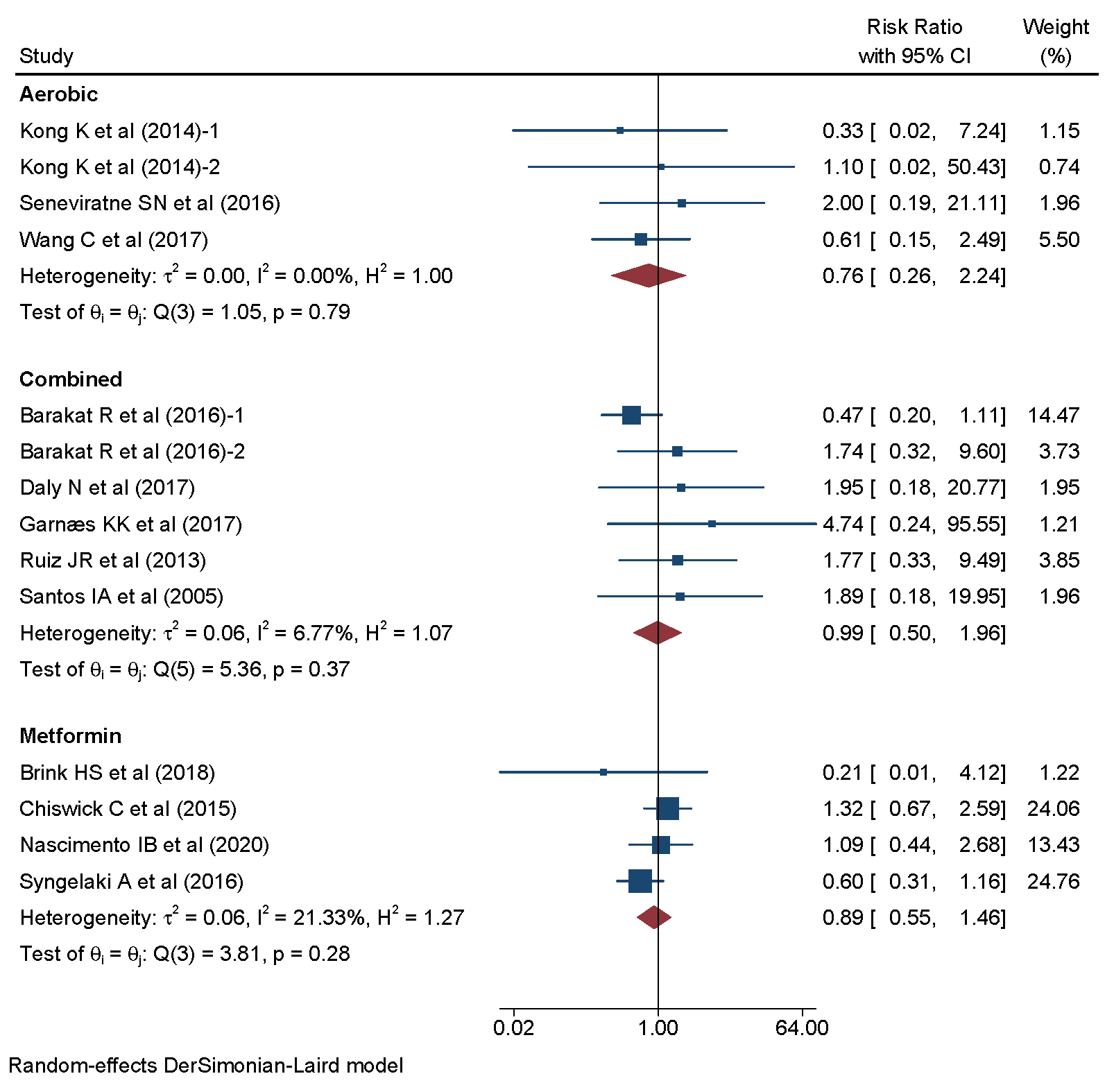


**C.** Macrosomia


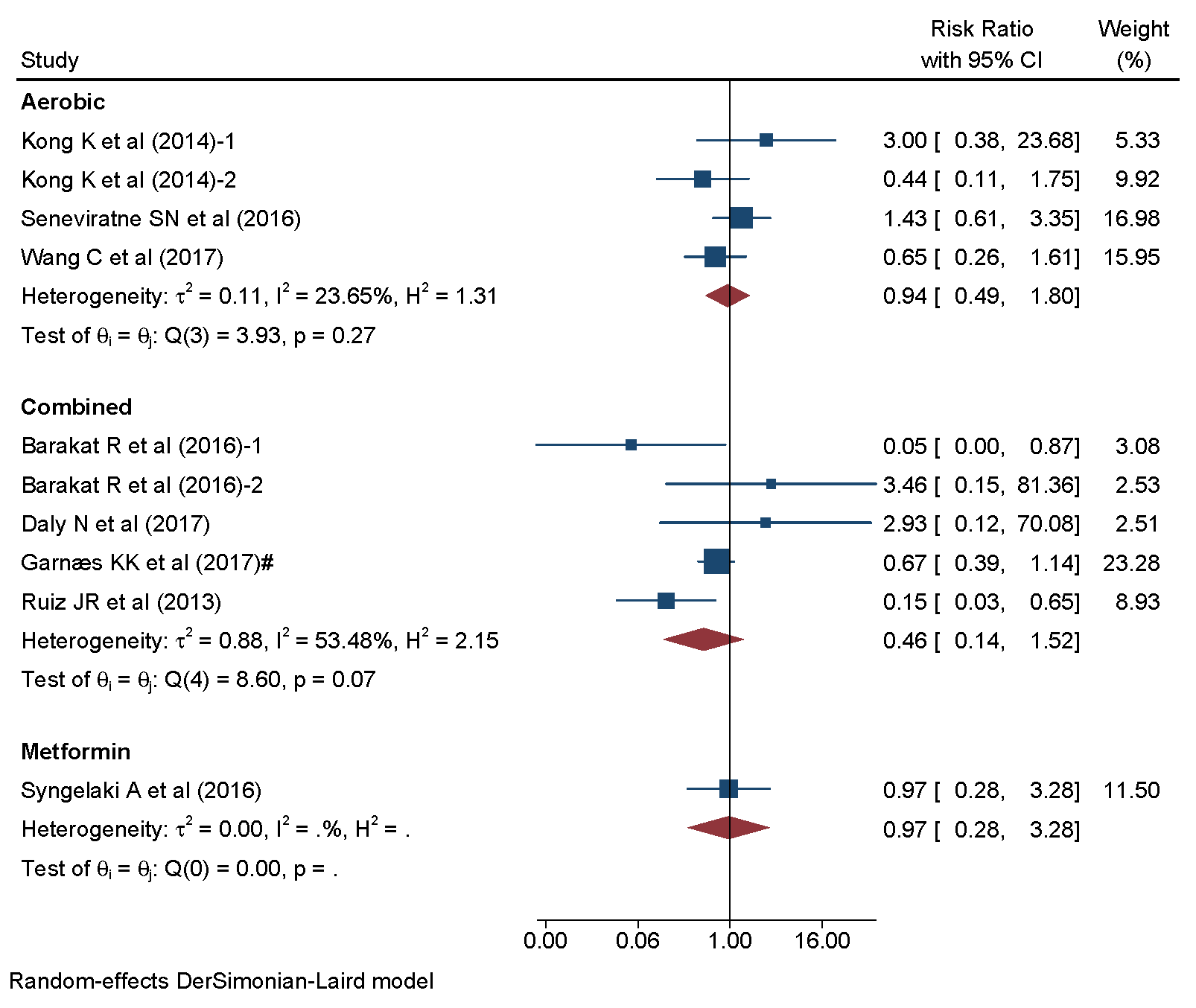


**D.** Birth weight


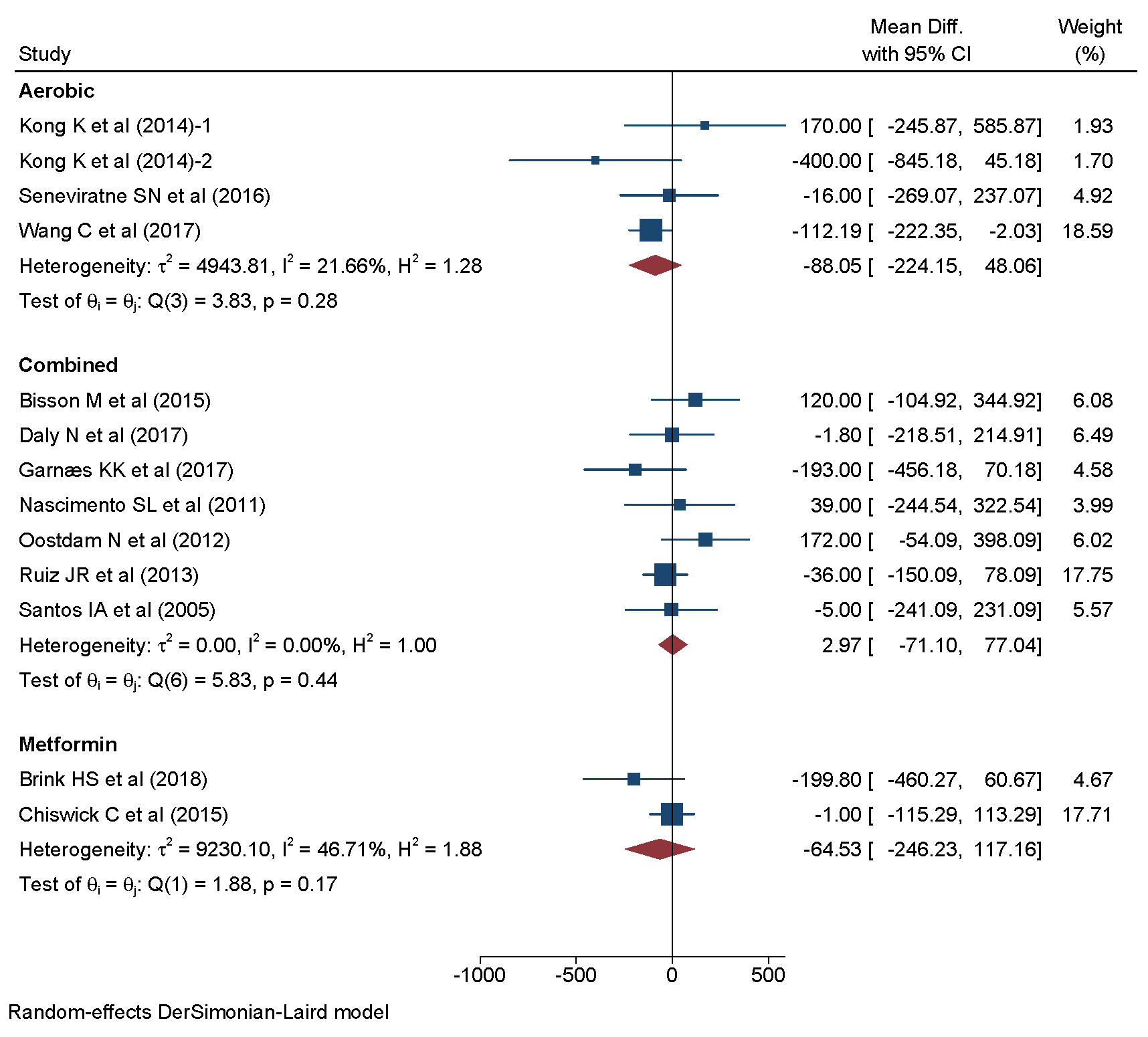


**A.** Caesarean section


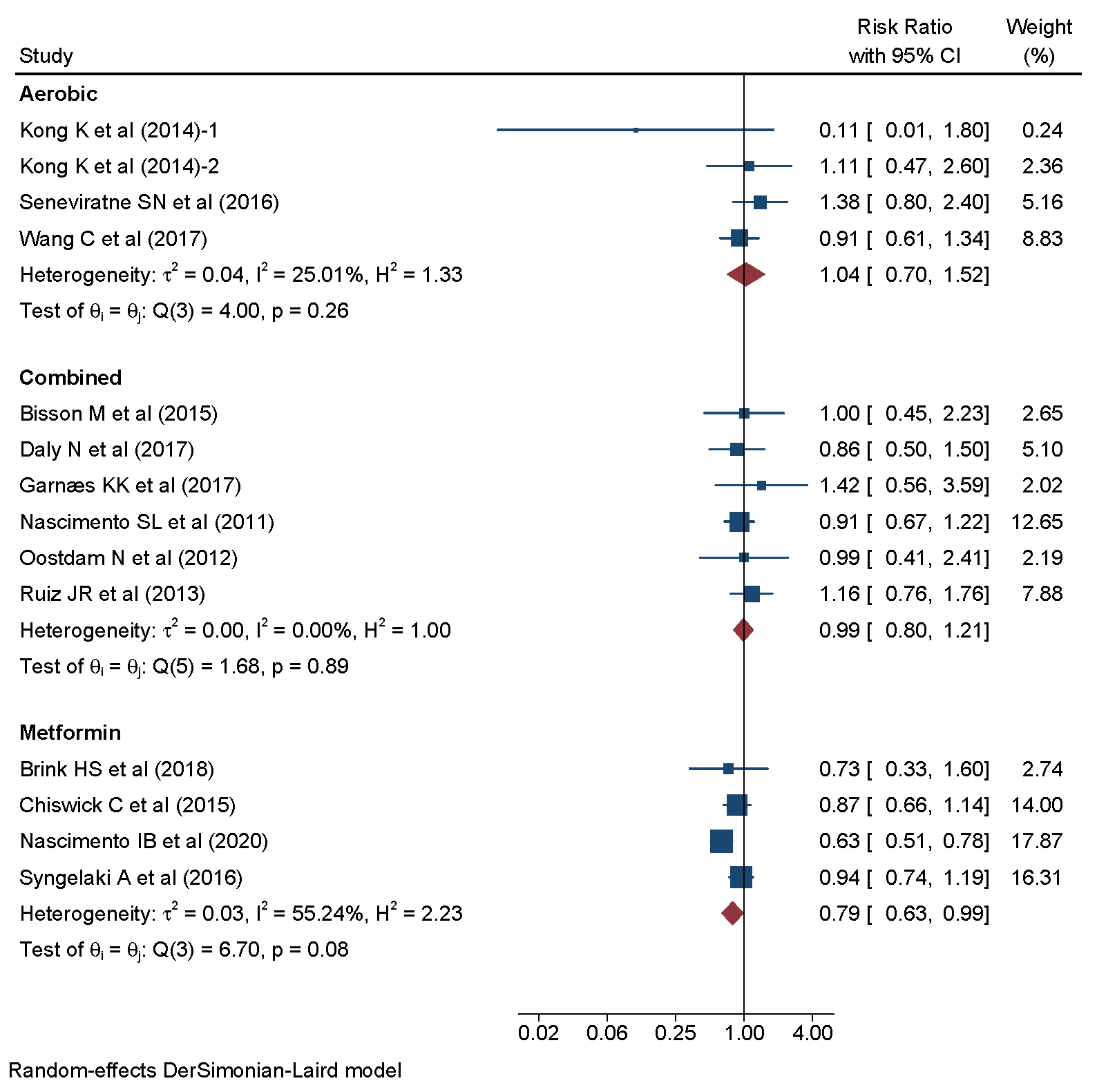


Random meta-analysis by outcome among women with overweight

**Figure S2.** Risk of bias.

**B.** Risk of bias graph


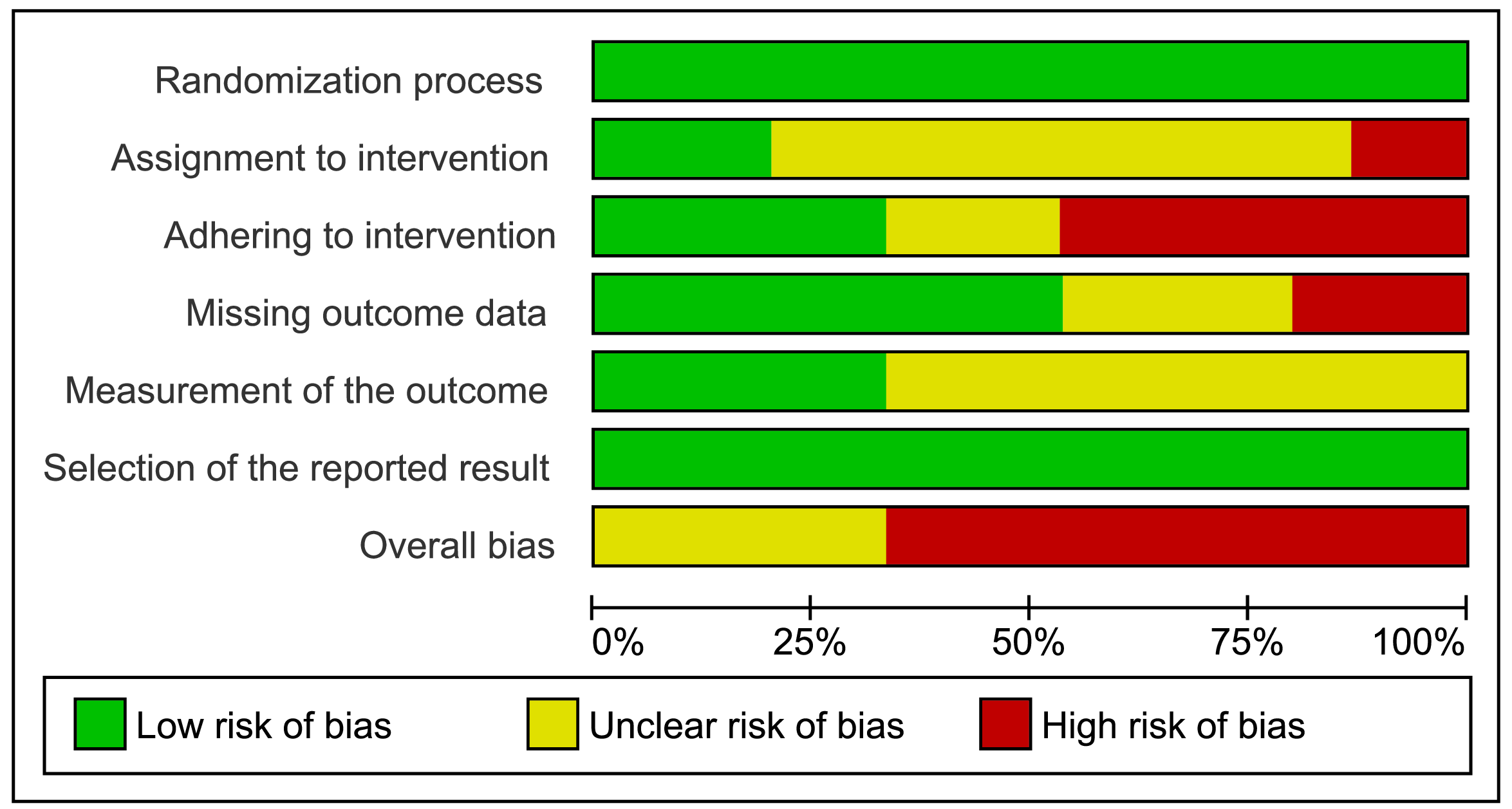


**A.** Risk of bias summary


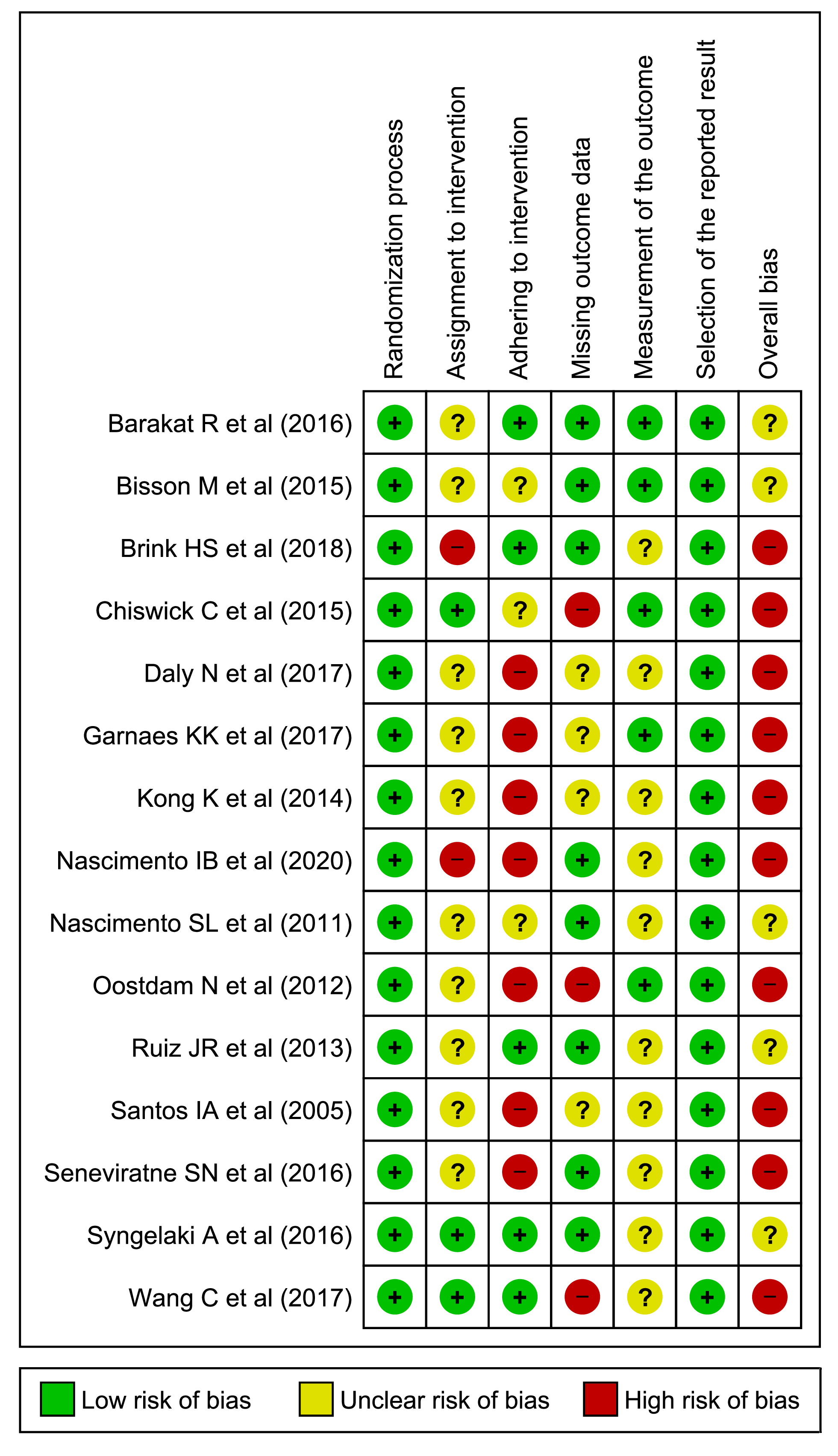


Detailed risk of bias for each study and domain (left), and global graph.

**Figure S3.** Cumulative probabilities by outcome.

**C.** Macrosomia


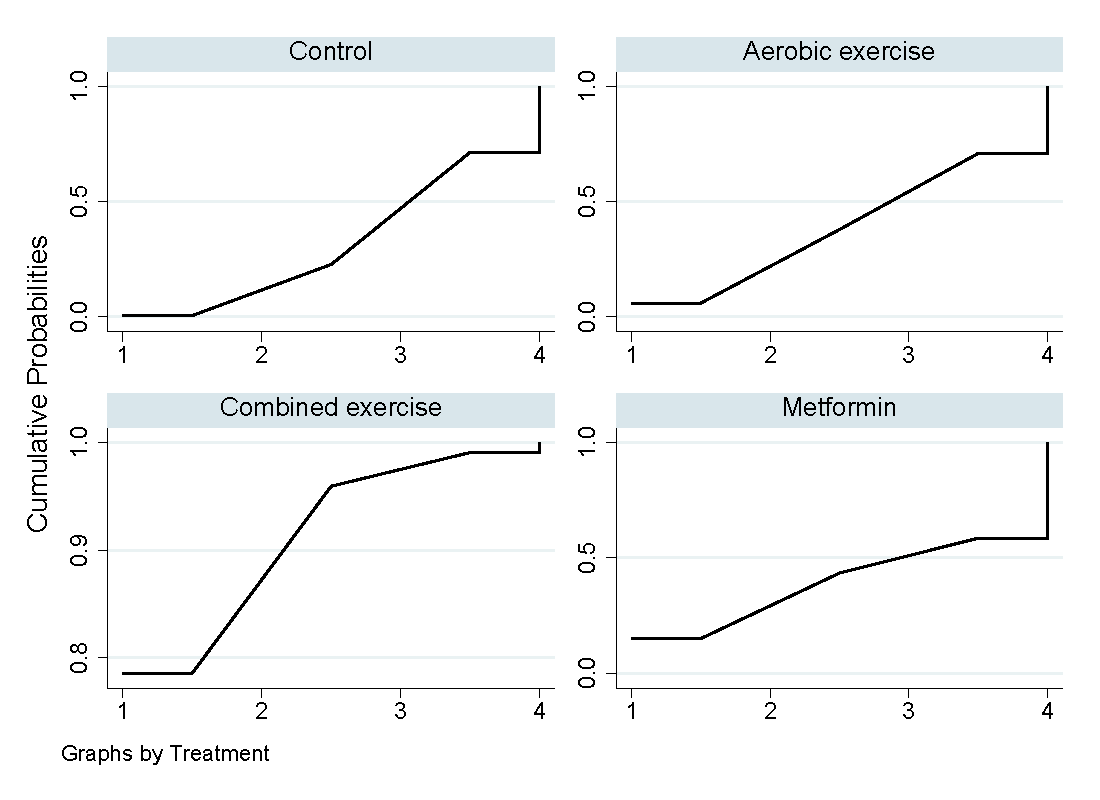


**D.** Birth weight


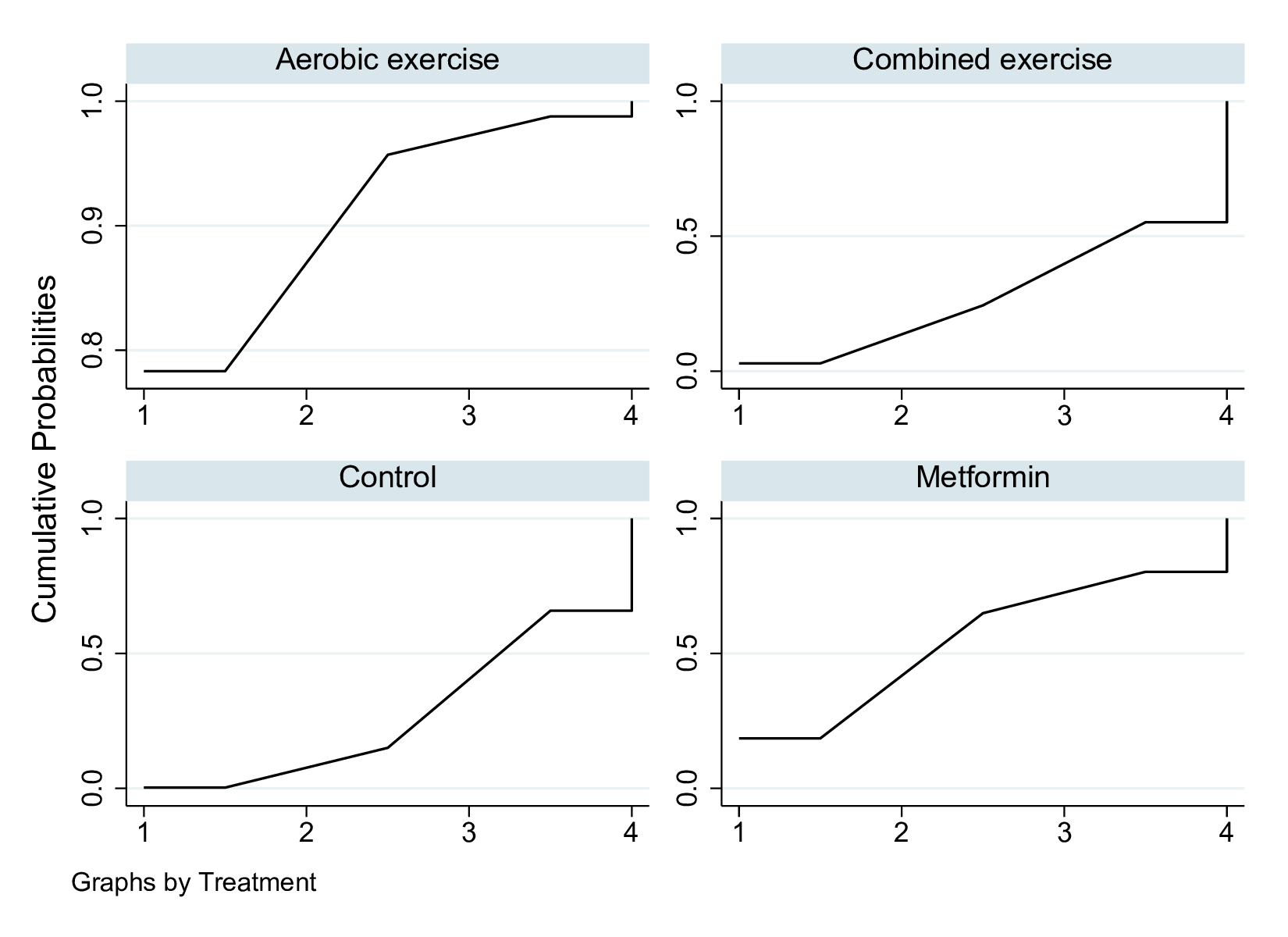


**B.** Preterm birth


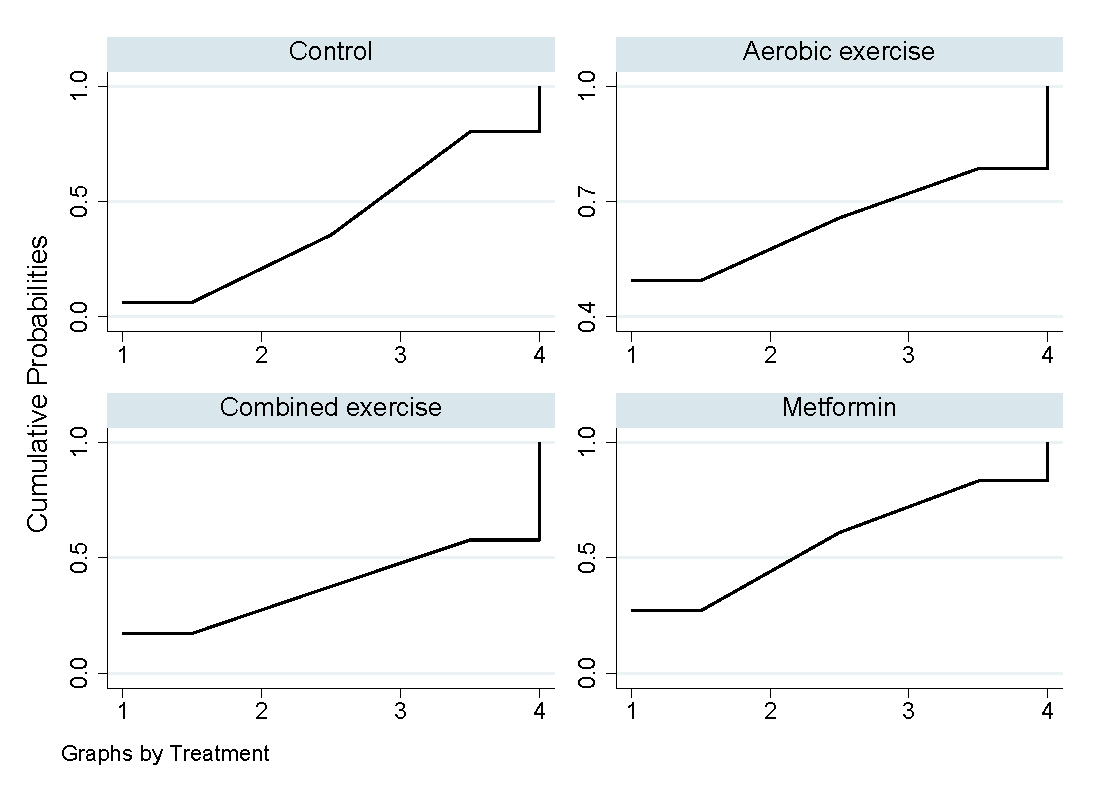


**A.** Caesarean section
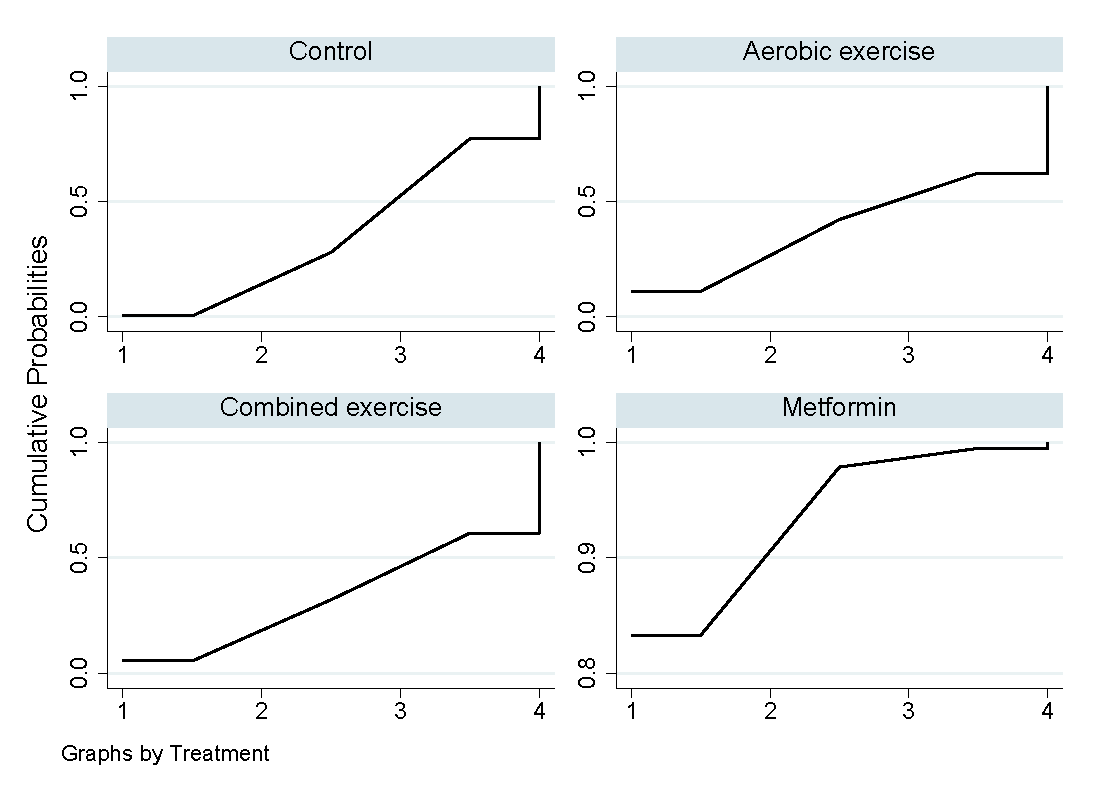


Cumulative probabilities, by outcome and probability of being the most effective intervention.

**Figure S4.** Direct pairwise comparisons and network meta-analysis by outcome among pregnant women with obesity.


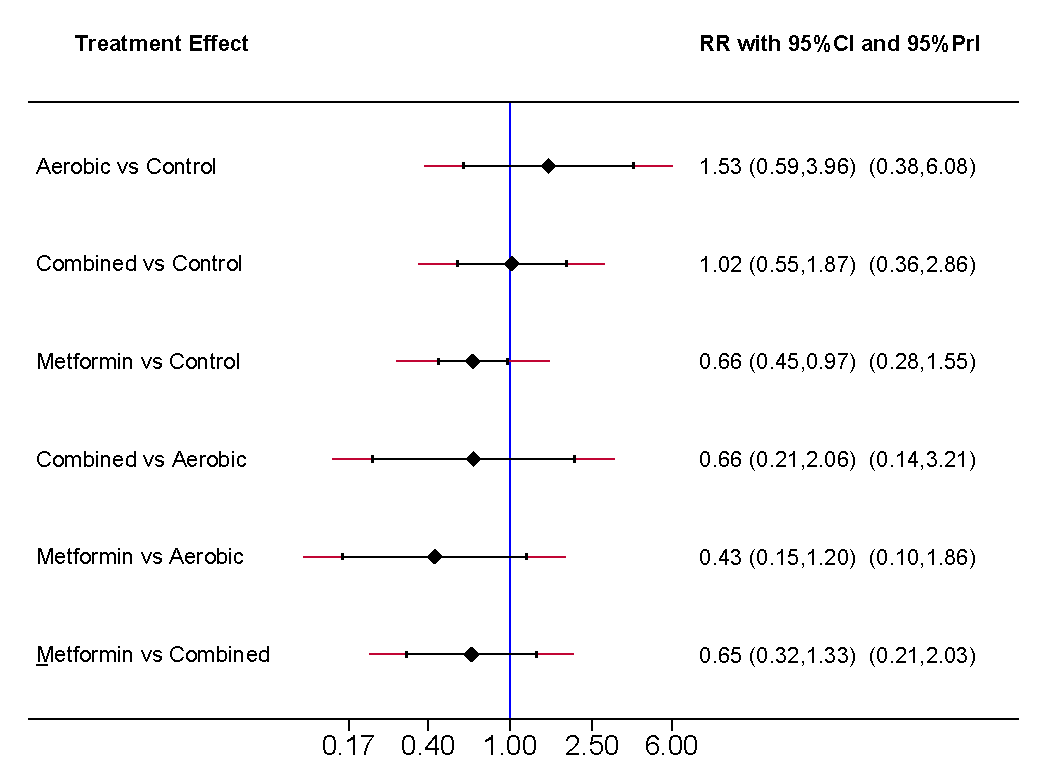
**A.** Caesarean section


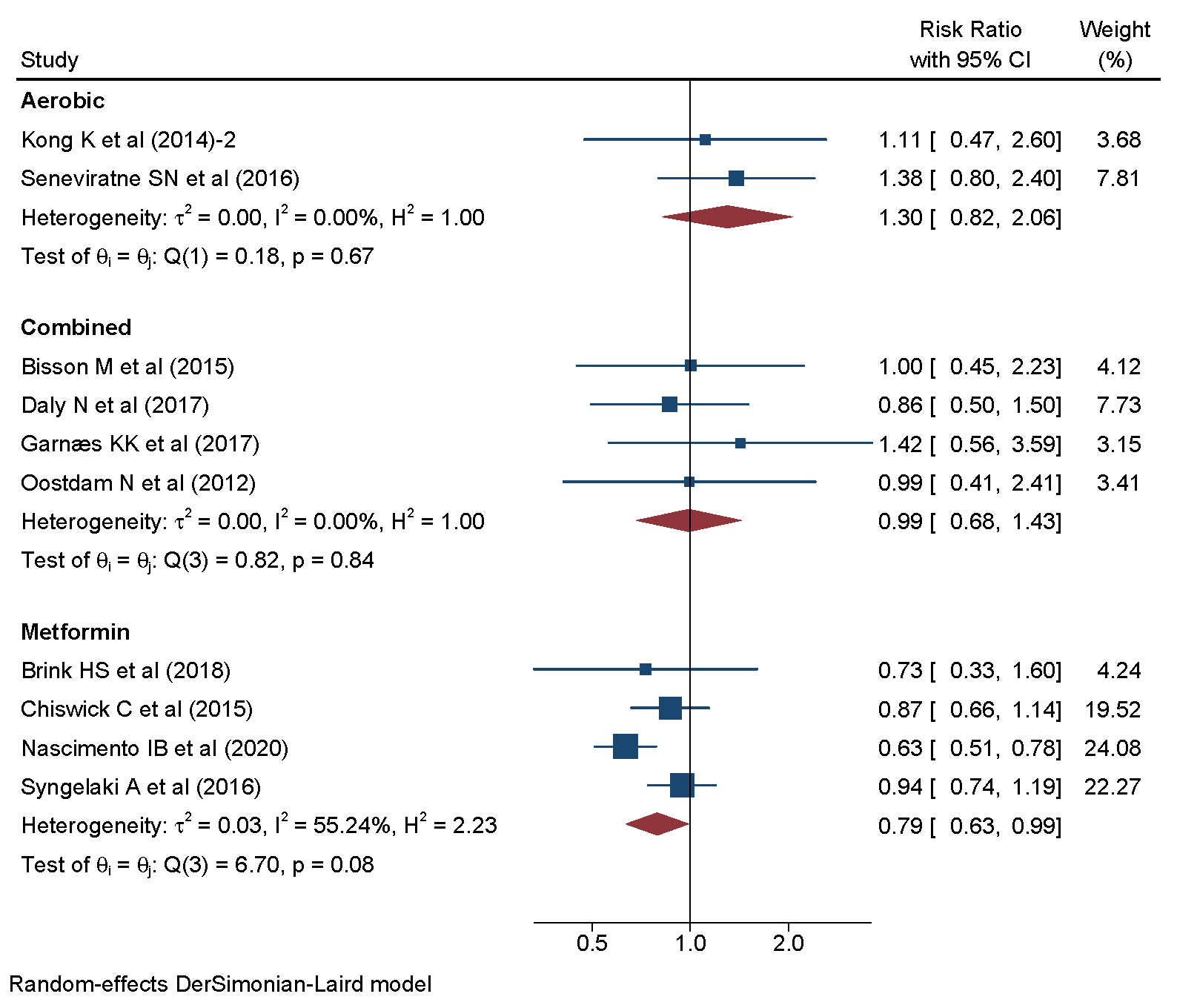


**B.** Preterm birth


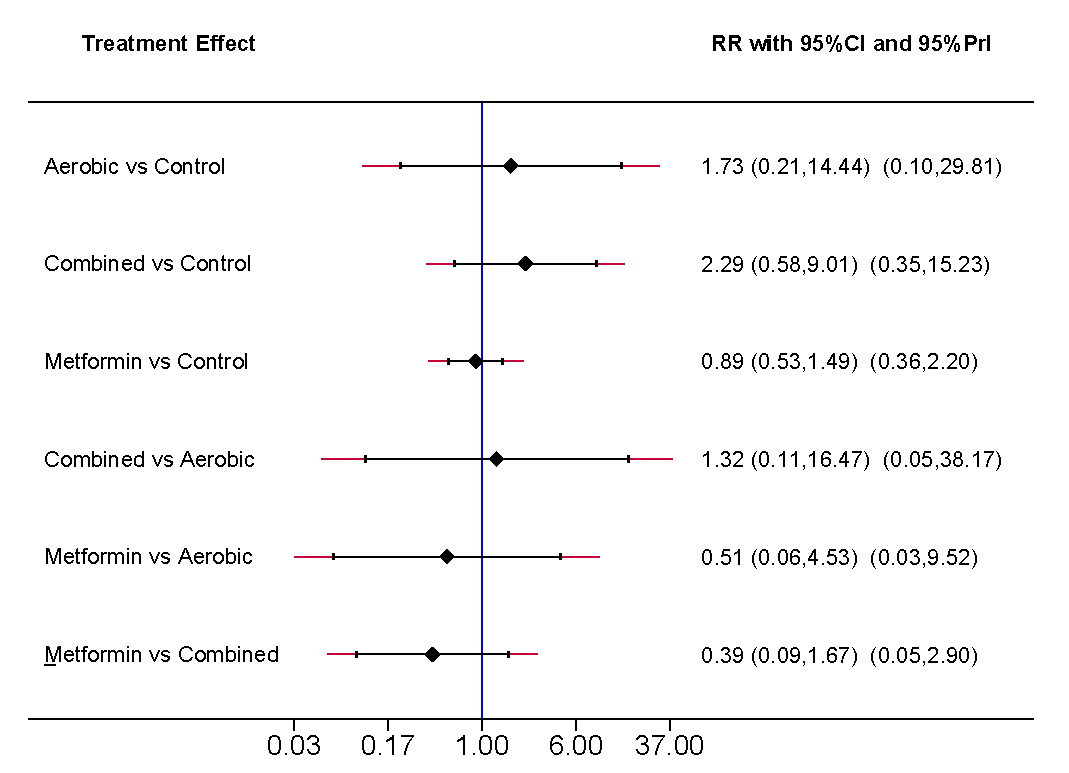

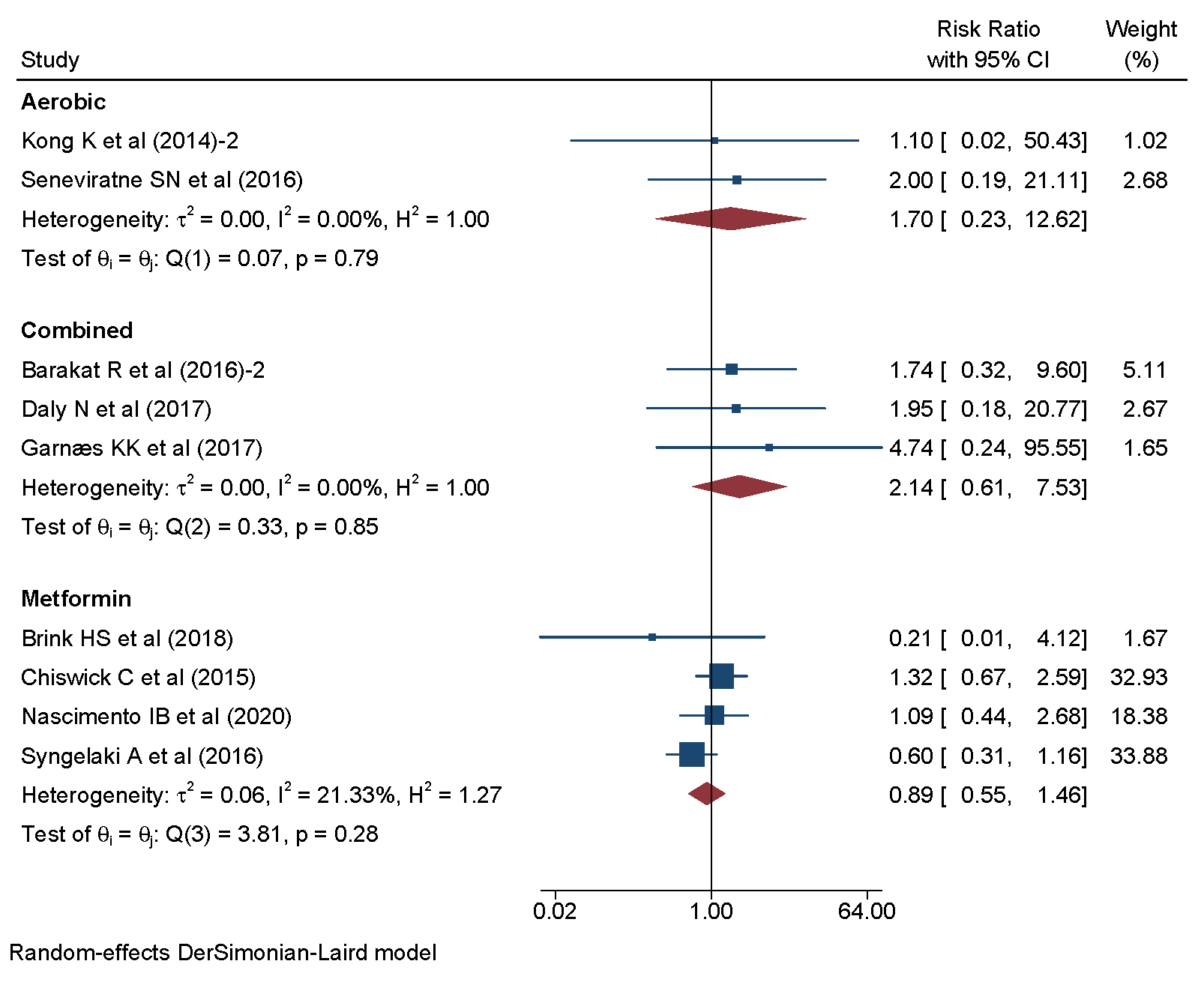


**C.** Macrosomia


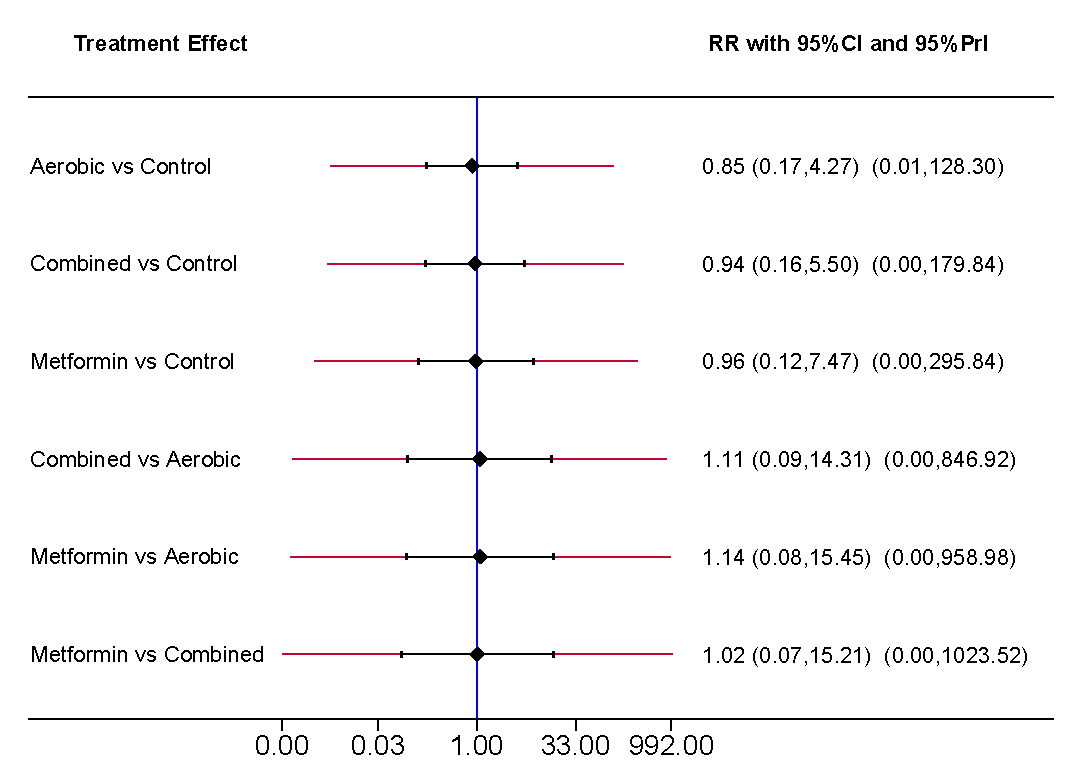

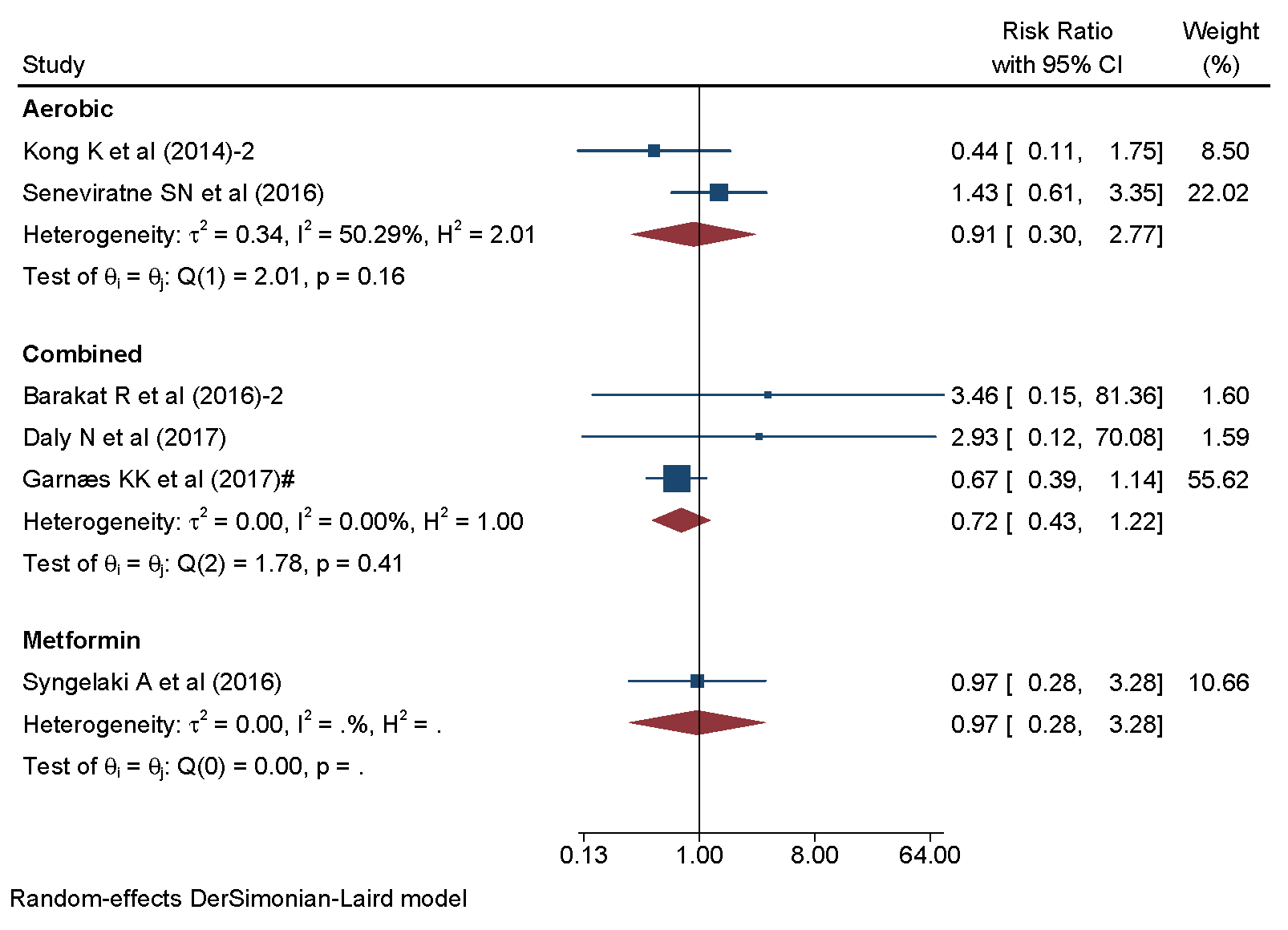


**D.** Birth weight


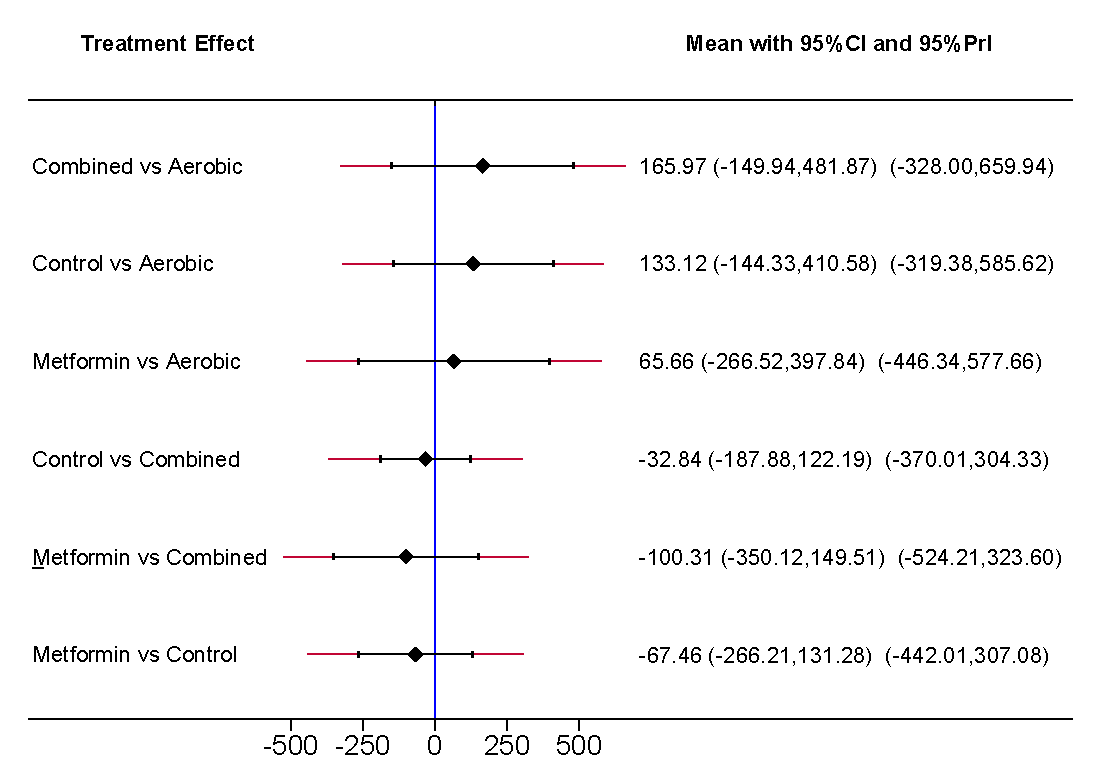

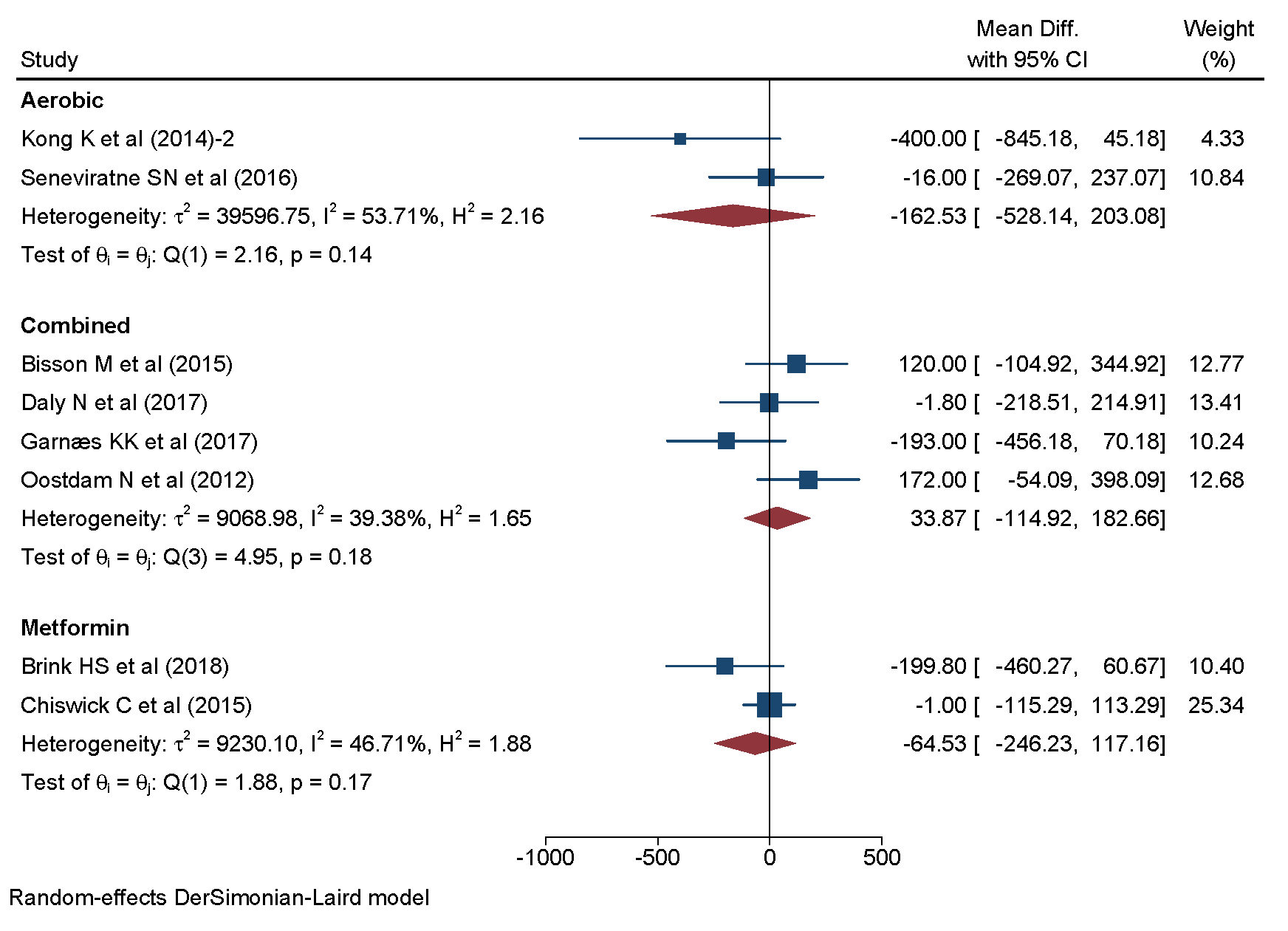


Random meta-analysis by outcome among women with obesity

**Figure S5.** Funnel plot by outcome.

**C.** Macrosomia


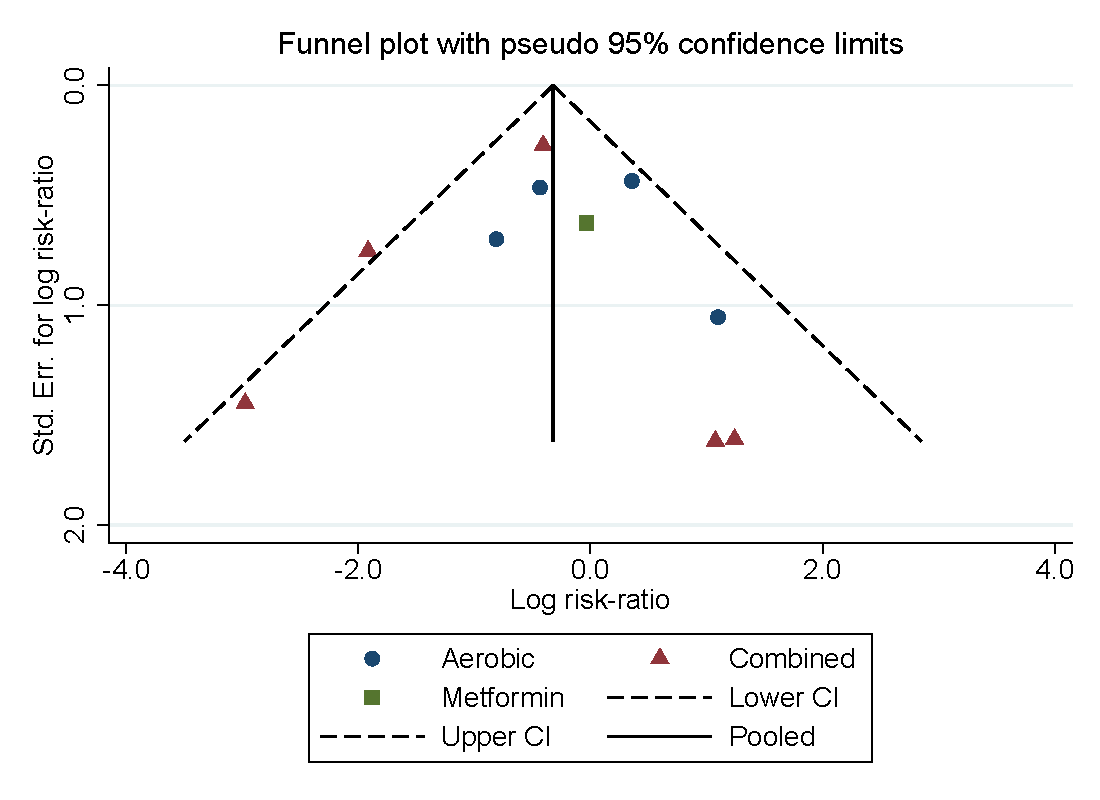


**B.** Preterm birth


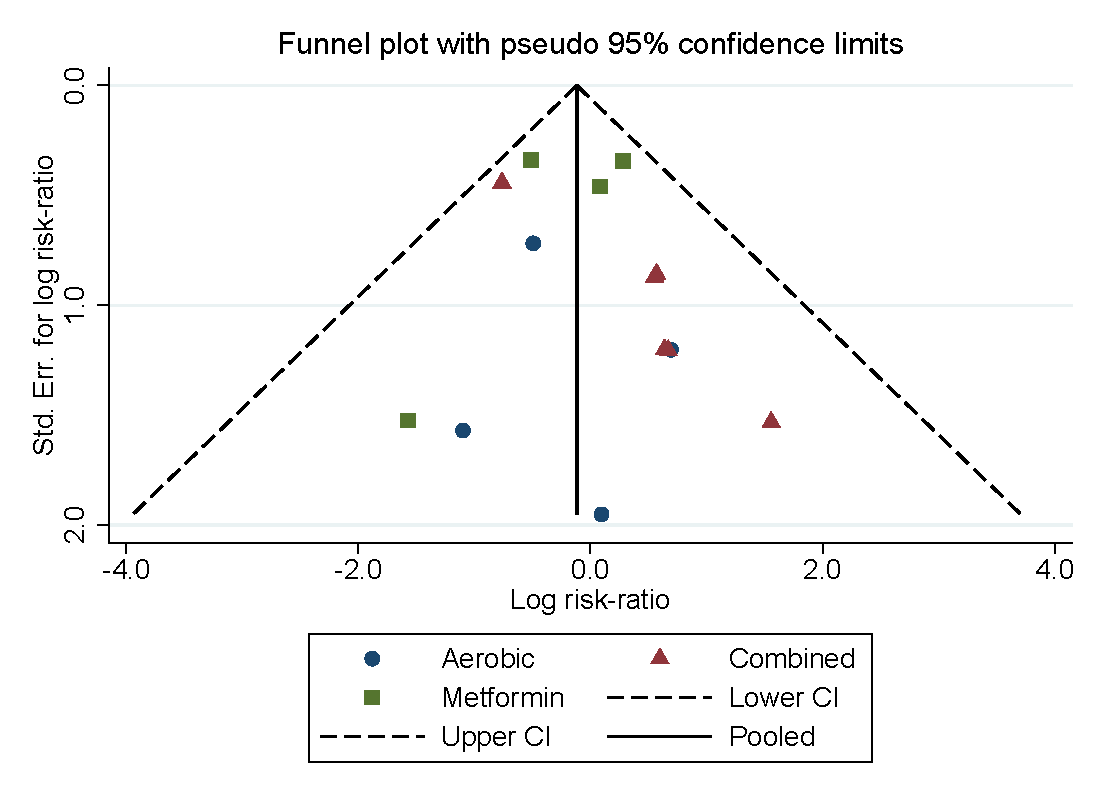


**A.** Caesarean section


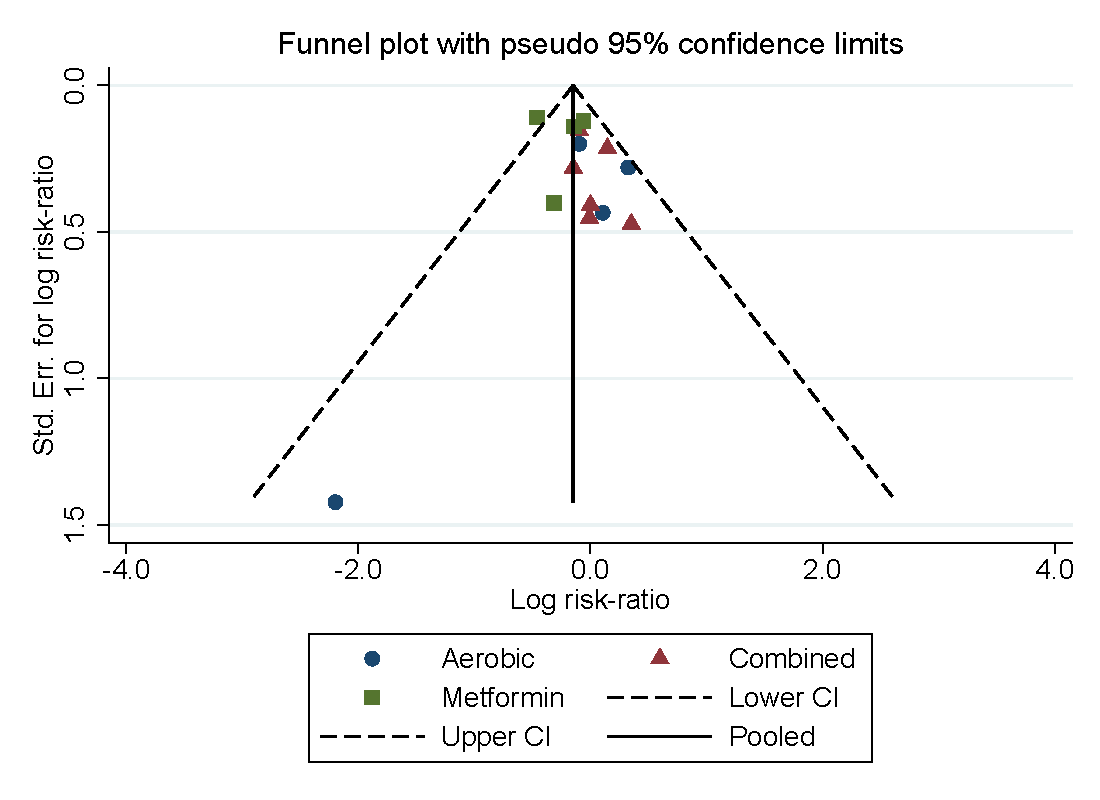


**D.** Birth weight


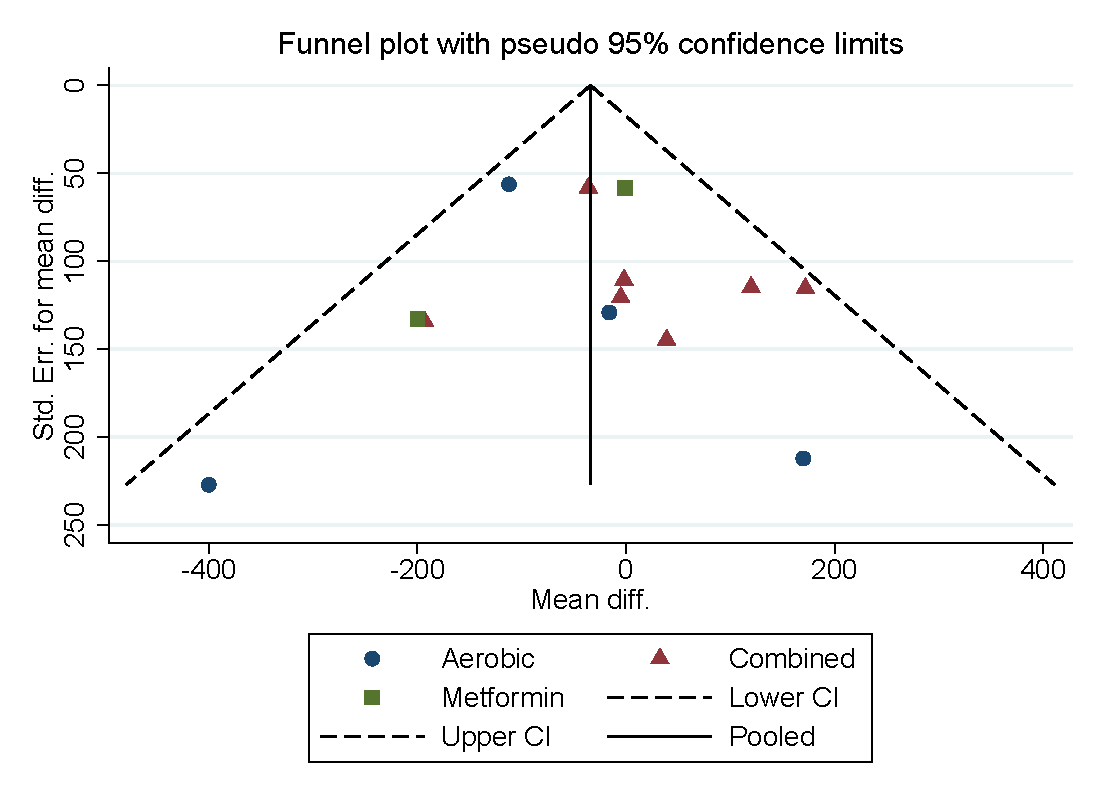


Funnel plots by outcome and their 95% confidence interval

**Appendix S1.** Search Strategy.

- Medline, Scopus, Web of Science, Cochrane Library:

(“pregnant” OR “pregnancy”) AND (“physical activity” OR “physical exercise” OR “exercise” OR “metformin”) AND (“trial” OR “randomized control trial” OR “randomized controlled trial” OR “controlled pre-post study”)

- NCT Trials:

Condition or disease: pregnancy; Other terms: physical activity

Condition or disease: pregnancy; Other terms: physical exercise

Condition or disease: pregnancy; Other terms: metformin

- EudraCT:

(“pregnancy” OR “pregnant”) AND (“physical activity” OR “physical exercise” OR “exercise” OR “metformin”)

- Other databases

Open search (not specified)
